# A novel chaotic and neighborhood search-based artificial bee colony algorithm for solving optimization problems

**DOI:** 10.1038/s41598-023-44770-8

**Published:** 2023-11-22

**Authors:** Wen-sheng Xiao, Guang-xin Li, Chao Liu, Li-ping Tan

**Affiliations:** 1 National Engineering Laboratory of Offshore Geophysical and Exploration Equipment, China University of Petroleum (East China), Qingdao, 266580 China; 2 School of Electrical and Mechanical Engineering, China University of Petroleum (East China), Qingdao, 266580 China

**Keywords:** Engineering, Mathematics and computing

## Abstract

With the development of artificial intelligence, numerous researchers are attracted to study new heuristic algorithms and improve traditional algorithms. Artificial bee colony (ABC) algorithm is a swarm intelligence optimization algorithm inspired by the foraging behavior of honeybees, which is one of the most widely applied methods to solve optimization problems. However, the traditional ABC has some shortcomings such as under-exploitation and slow convergence, etc. In this study, a novel variant of ABC named chaotic and neighborhood search-based ABC algorithm (CNSABC) is proposed. The CNSABC contains three improved mechanisms, including Bernoulli chaotic mapping with mutual exclusion mechanism, neighborhood search mechanism with compression factor, and sustained bees. In detail, Bernoulli chaotic mapping with mutual exclusion mechanism is introduced to enhance the diversity and the exploration ability. To enhance the convergence efficiency and exploitation capability of the algorithm, the neighborhood search mechanism with compression factor and sustained bees are presented. Subsequently, a series of experiments are conducted to verify the effectiveness of the three presented mechanisms and the superiority of the proposed CNSABC, the results demonstrate that the proposed CNSABC has better convergence efficiency and search ability. Finally, the CNSABC is applied to solve two engineering optimization problems, experimental results show that CNSABC can produce satisfactory solutions.

## Introduction

Optimization problems have a large number of applications in many fields, such as engineering design, optimization of structural parameters, financial investments, etc.^1,2^. To improve the handling of these problems, a series of global optimization algorithms have been developed to traditional mathematical theories and solution methods^3,4^, which are divided into deterministic optimization algorithms and stochastic optimization algorithms^5^. As stochastic optimization algorithms, metaheuristic optimization algorithms have high solution accuracy and efficiency, mainly including genetic algorithm (GA) inspired by biological evolution^6^, simulated annealing algorithm (SA)^7^ and gravity search algorithm (GSA) inspired by physical principles^8^, particle swarm algorithm (PSO) inspired by animal population behavior^9^ and artificial bee colony algorithm (ABC)^10^. As optimization problems become more and more complex, many metaheuristic optimization algorithms have been presented to solve large-scale global optimization (LSGO) problems, such as the wild goose algorithm (WGA)^11^, the african condor optimization algorithm (AVOA)^12^, the australian wild dog optimization algorithm (DOA)^13^. Conscious neighborhood-based crow search algorithm (CCSA)^14^, starling murmuration optimizer (SMO)^15^, diversity-maintained multi-trial differential evolution algorithm (DMDE)^16^ and enhanced moth-flame optimization algorithm using an effective stagnation finding and replacing strategy (MFP-SFR)^17^, etc.

Compared with the other metaheuristic algorithms, the ABC has advantages such as few control parameters, easy implementation, and outstanding exploration capability, it performs both global and local optimal solution searches during each iteration, therefore the probability of being able to find the optimal solution is greatly increased.

The ABC is a novel swarm intelligence algorithm by simulating the foraging behavior of bees, which is widely used in the fields of PID parameter optimization, image processing, numerical optimization, structural design, etc. Bingul et al.^18^ compared the PSO and the ABC to find the best performance parameters of the PID controller, and the results of the robustness analysis show that the PID controller parameters adjusted by the ABC have stronger robustness under the internal and external perturbations. Öztürk et al.^19^ analyzed the improved ABC for medical image processing proposed during 2010–2020. Hussain et al.^20^ proposed the improved ABC for copolymerization of high-dimensional data, and showed that the combination of a new similarity measure and an optimized local search method, significant progress was achieved in searching for optimal clusters. Sagayam et al.^21^ proposed a hybrid one-dimensional HMM model with ABC to optimize its parameters and observed state sequences to improve performance, and the results showed a very low recognition error rate. Li et al.^22^ proposed an ABC algorithm-based structural design optimization method for fiber-reinforced plastic (FRP) vessels, and the results showed that the weight of a 32.98 m FRP fishing vessel could be reduced by 8.31%.

However, the traditional ABC contains some disadvantages, such as slow convergence, easy stagnation, etc. Therefore, many researchers delivered a lot of improvement measures to enhance the convergence speed and exploitation capability of ABC. Zhang et al.^23^ proposed an improved ABC algorithm with a unitary inheritance (OPI) mechanism (OPIABC), to address the fact that the solution in the ABC varies in only one dimension. Wang et al.^24^ presented a selection method based on the radius of the neighborhood, which improves the search phase of the detection bee and enhances the exploitation of the ABC. Shi et al.^25^ introduced the concept of queen bee to propose a new neighborhood search mechanism and improved the dimensional selection strategy to realize the conversion between one-dimensional search and full-dimensional search. In real bee colonies, onlooker bees and employed bees have different exploitation mechanisms, and onlooker bees choose the best one nectar source for exploitation. Karaboga et al.^26^ designed a new search equation for onlooker bees, and the proposed quick artificial bee colony algorithm (qABC) more accurately simulates the behavior of onlooker bees and improves the local search capability of the ABC. To enhance the global convergence speed, Gao et al.^27^ introduced a new search mechanism which include logistic chaos mechanism and backward learning to improve algorithm, the mechanism can control the probability of introducing two search equations. Xiao et al.^28^ proposed a new adaptive neighborhood search gaussian perturbation algorithm (NGABC), which first used an adaptive method to adjust the neighborhood, then applied the global optimal solution to guide the search, and finally designed a new Gaussian perturbation.

To solve the shortage of ABC algorithm with strong exploration capability and weak development capability, Zhu et al.^29^ proposed the Gbest-guided artificial bee colony algorithm (GABC), which adds the influence of global optimal solution to the neighborhood search equation and improves the exploitation capability of the algorithm. Zheng et al.^30^ used cat chaos mapping to increase the diversity of the solutions in the initial stage, applied differential evolution to improve the search strategy, and designed adaptive scaling factors to achieve dynamic search. To improve the development efficiency and convergence speed, Chouaib et al.^31^ proposed a multiple population ABC based on global and local optima (MPGABC), which divides the population into multiple subpopulations and introduces global and local optimal solutions in the search equation of the solution. Brajevi´ et al.^32^ added shuffle variation operators to the hiring bee and onlooker bee phases, making the algorithm get a good balance between global search ability and local exploitation ability to solve integer programming and minimax problems. Zhao et al.^33^ proposed a novel method (QABC) with a search equation based on the idea of quasi-Avanti transformation, which enhanced the exploitation capability of the algorithm, and then introduced a collaborative search matrix to update the position of the nectar source to ensure the randomness and balance of the search. Even though the improved ABC algorithms can produce satisfying solutions in solving optimization problems, with regard to effectiveness and efficiency (such as slow convergence speed, easy premature maturation), there is still space for improvement further of ABC performance.

In this study, a novel chaotic and neighborhood search-based artificial bee colony algorithm (CNSABC) is proposed. The proposed CNSABC includes three novel mechanisms, which are chaotic mapping with mutual exclusion mechanism, neighborhood search mechanism with compression factor and sustained bees. The chaotic mapping with mutual exclusion mechanism is introduced to have better ergodicity in the solution space and enhance global exploration; the neighborhood search mechanism with compression factor is presented to and enhance the convergence efficiency and local exploitation capability. A new type of bee named sustained bees is proposed to improve the ability to explore optimal solution, further to avoid the appearance of premature maturity in some degree. These three strategies work together to improve the performance of global exploration and local exploitation, resulting in a faster convergence speed and decent quality of solutions for the ABC. To verify the performance of CNSABC and confirm the effectiveness of the proposed mechanisms, three sets of numerical experiments are conducted on selected 26 benchmark functions. The first set includes CNSABC and the ABC algorithm with a single strategy for improvement, the second set includes CNSABC and five commonly used metaheuristic optimization algorithms (ABC, PSO, GWO^34^, WOA^35^, and BOA^36^), the last set includes CNSABC and five improved ABC algorithms in other literatures (qABC, SBABC, MPGABC, GABC, and NGABC). In addition, the Tension/compression spring design problem and the Speed reducer design problem are used to test the ability of the CNSABC for solving real engineering problems. The results verify the dominant of CNSABC with regard to the convergence speed and optimal solution search ability, which can also indicate the three proposed mechanisms play a guiding role on enhancing ABC algorithm.

The rest sections of this study are arranged as follows. Section "Improved ABC algorithm" introduces the principle and pseudo-code of the ABC algorithm and the strategy of the CNSABC in detail. Section "Experimental results and analyses" shows three sets of experimental results comparing the CNSABC with other algorithms and analyzes the effect of the improvements. Section "CNSABC for solving engineering optimization problems" uses two engineering example problems to verify the practicality of CNSABC for solving practical problems. Section "Conclusions" summarizes this research and illustrates some future research directions.

## Improved ABC algorithm

### Traditional ABC algorithm

**Figure 1:**
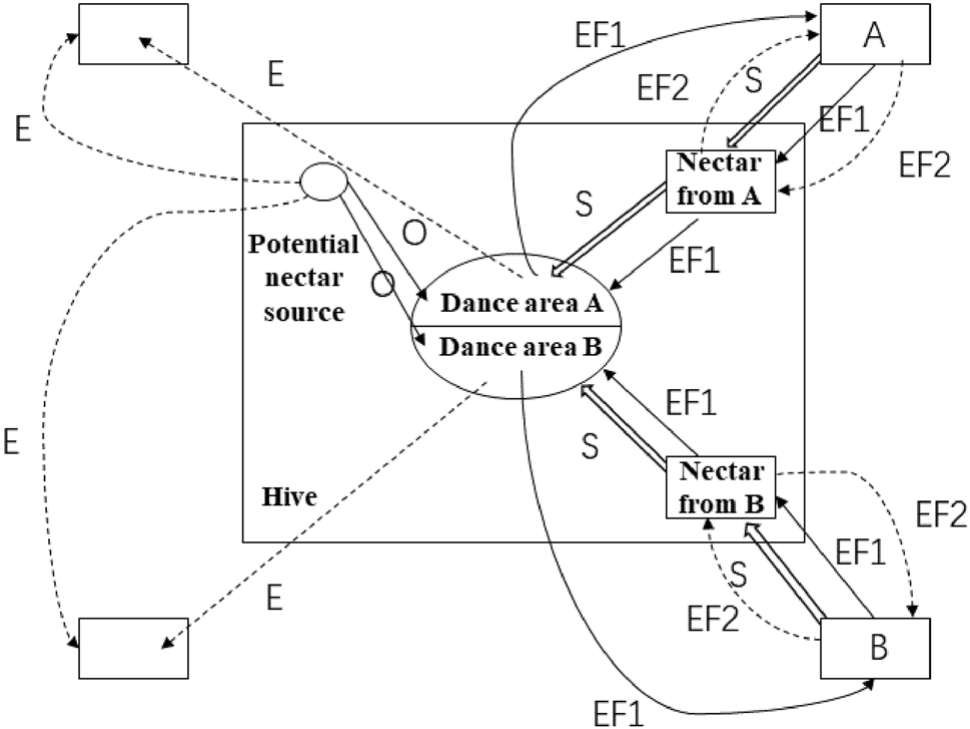
Honey foraging behavior.

ABC algorithm is inspired by the foraging behavior of honeybees. The foraging behavior of bees is shown in Fig. 1. In a colony, there are three main types of bees: employed bees (E), onlooker bees (O), and scout bees (S). Each bee is closely related to the location of the nectar source. Employed bees harvest nectar near the initial nectar source (A, B) and share nectar information (EF1) within the hive (Dance area A, B), onlooker bees select some better nectar sources to exploit (EF2), and when the nectar source has no nectar, onlooker bees transform into scout bees to find new nectar sources. ABC algorithm is an iterative process and the steps of the algorithm are as follows.

*Initialization* The nectar source in the ABC is a multidimensional vector. ABC starts the search from a set of randomly distributed initial nectar sources, which are generated by using Eq. (1).

1 $$x_{ij} = x_{j}^{\min } + rand \times \left( {x_{j}^{\max } - x_{j}^{\min } } \right)$$

in which, *i* and *j* take values in the interval [1, *SN*] and [1, *D*], $x_{j}^{\min }$ and $x_{j}^{\max }$ are the lower and upper limits of the *j-*th dimension, *SN* is the total number of nectar sources, *D* is the number of dimensions, and *rand* is a uniform random number in the value interval [0, 1].

*Employed bees phase* Each employed bee is associated with a nectar source ($x_{ij}$), and performs a neighborhood search in the vicinity of the associated nectar source, which in turn produces a new nectar source ($v_{ij}$), the location is generated by using Eq. (2).

2 $$v_{ij} = x_{ij} + \phi_{ij} \times \left( {x_{ij} - x_{kj} } \right)$$

in which, *i* is the serial number of the current honey source, *k* is the serial number of other honey sources, *k* ∈ [1, SN], *k* ≠ *i*, $\phi_{ij}$ is a random number within the value interval of [− 1, 1]. In this phase, the employed bee compares the original nectar source with the new one and then chooses the better one to develop and returns to the hive to share the information of the better nectar source with other bees.

*Onlooker bees phase* Onlooker bees evaluate all known honey sources and select a certain probability of nectar source for exploitation, the probability ($p_{i}$) of nectar source selection is calculated by using Eq. (3).

3 $$p_{i} = \frac{{fit_{i} }}{{\sum\nolimits_{j} {fit_{j} } }}$$

in which, $fit_{i}$ is the fitness value of the *i-*th nectar source. The nectar source with better fitness value is more likely to be selected and then follow the bee to exploit this source and then update the nectar location by Eq. (2). Like the employed bee, the better nectar source will be selected for retention based on the greed criterion.

*Scout bee phase* Employed bees and onlookers may keep taking nectar from an un-renewed nectar source, therefore an upper limit is set on the number of times a nectar source can be exploited. When the upper limit of exploitation is reached and the nectar source is not renewed, this source is abandoned, and the bees that were following it are transformed into scout bees to regenerate a new source using Eq. (1).

### The proposed CNSABC

#### Bernoulli chaotic mapping with mutual exclusion mechanism

The initial solution of the traditional ABC is generated by using Eq. (1), where *rand* is a uniformly distributed random number belongs to the pseudo-random number^37–41^. However, when solving high-dimensional problems, the initial population generated in this way is not uniform enough. Therefore, it is not guaranteed to get a better population in the global search. In addition, the use of *rand* in the search process reduces the local search ability in the employed bees phase and onlooker bees phase. To overcome this shortcoming, a chaotic mapping with mutual exclusion mechanism is introduced to generate the initial population.

**Figure 2:**
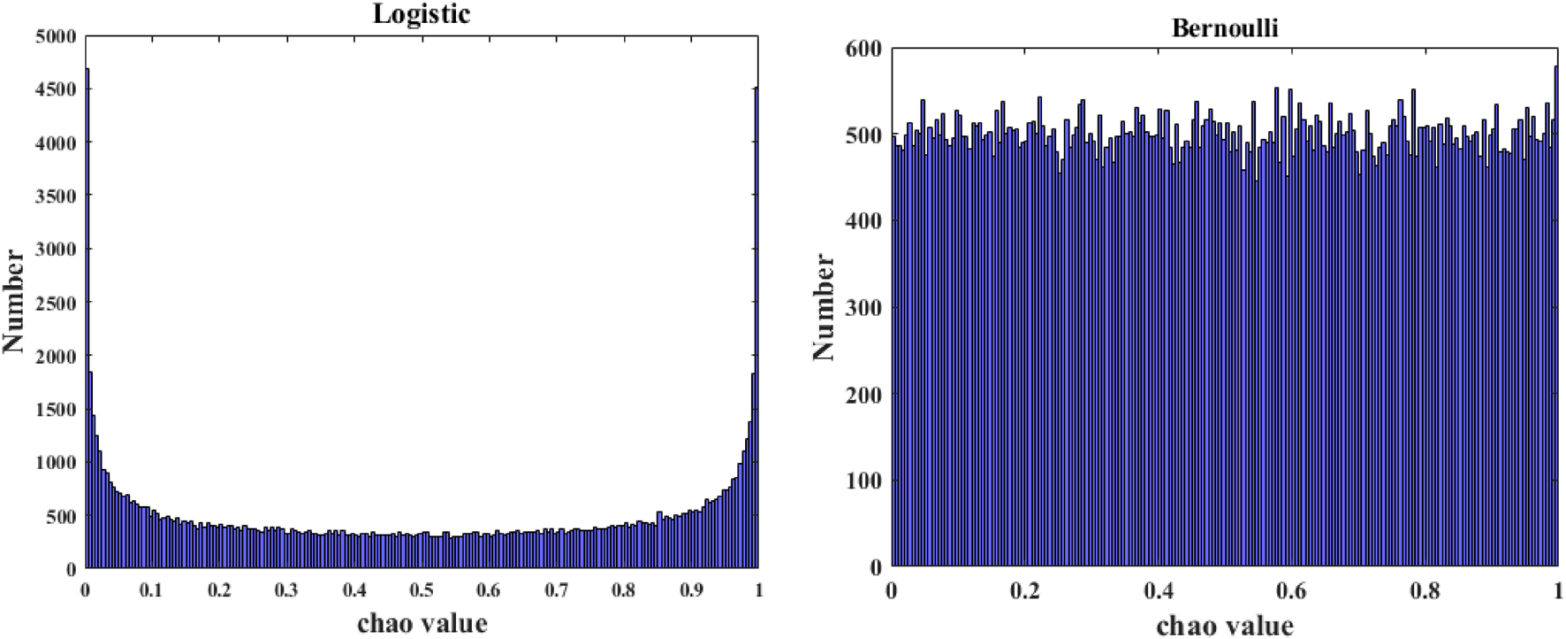
The chaos number distribution.

Chaos mapping methods mainly include Logistic chaotic mapping and Bernoulli chaotic mapping^42–44^. Among them, the Logistic chaotic mapping is the most widely used. However, the logistic chaotic mapping has a high probability of taking values in the interval [0, 0.1] and [0.9, 1], which is not uniformly traversed in the global optimization search process and may lead to reduce the efficiency of the algorithm. Bernoulli chaos mapping is uniformly distributed between [0, 1]. Compared with Logistic chaos mapping, Bernoulli chaos mapping has better traversal uniformity and randomness. The chaos value distribution of Logistic chaos mapping and Bernoulli chaos mapping at 10^5^ iterations is shown in Fig. 2.

The Bernoulli chaotic mapping is e expressed by Eq. (4).

4 $$z_{k + 1}^{B} = \left\{ {\begin{array}{*{20}l} {z_{k}^{B} /(1 - \beta ),} \hfill & {z_{k}^{B} \in \left( {0,1 - \beta } \right]} \hfill \\ {(z_{k}^{B} - 1 + \beta )/\beta ,} \hfill & {z_{k}^{B} \in \left( {1 - \beta ,1} \right]} \hfill \\ \end{array} } \right.$$

in which, the range of $\beta$ is (0,1). In the range of $\beta$, the system is in the chaotic state. By introducing the Bernoulli chaotic mapping with mutual exclusion mechanism into the initial population generation equation, Eq. (1) becomes into Eqs. (5) and (6). The initial individual $x_{ij}$ is considered to be the one with the best fitness, as shown in Eq. (7). The mutual exclusion mechanism makes the search direction into two opposite directions, which can improve the exploration capability.

5 $$x_{i1j} = x_{j}^{\min } + z^{B} (x_{j}^{\max } - x_{j}^{\min } )$$

6 $$x_{i2j} = x_{j}^{{{\text{m}} ax}} - z^{B} (x_{j}^{\max } - x_{j}^{\min } )$$

7 $$x_{ij} = \min (x_{i1j} ,x_{i2j} )$$

**Figure 3:**
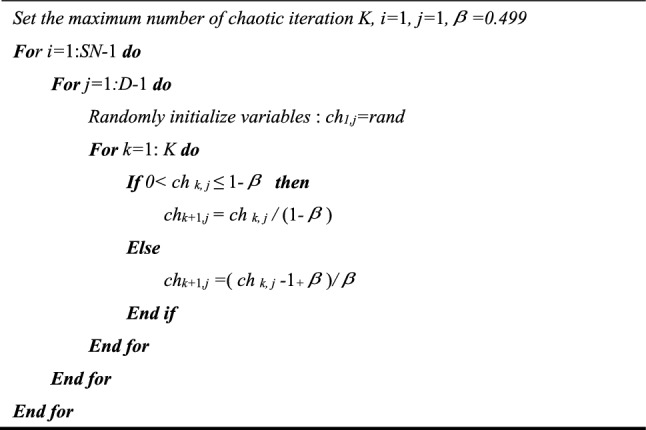
The pseudo-code of Bernoulli chaotic mapping.

The pseudo-code of Bernoulli chaotic mapping is shown in Fig. 3.

The standard deviation of the initial population individuals generated by Bernoulli chaos mapping with mutual exclusion mechanism, and the initial population individuals generated by *rand* are counted in the value range of [− 100, 100] to generate 30-dimensional, 50-dimensional and 100-dimensional population individuals, respectively. The larger the standard deviation of the generated initial population individuals, the better the initial population diversity. The standard deviation results are shown in Table1.

**Table 1:**
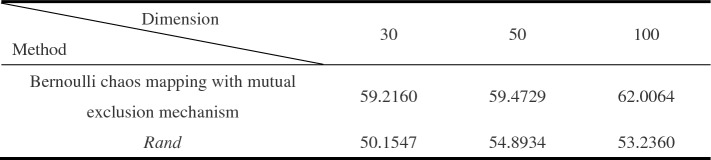
Standard deviation results.

#### Neighborhood search mechanism with compression factor

In nature, employed bees and onlooker bees play different roles in a bee colony. The main purpose of employed bees is to explore more nectar sources, and onlooker bees is to exploit known nectar sources. However, in traditional ABC algorithm, the same neighborhood search way is used for both employed and onlooker bees to simulate nectar collection behavior. This way leads to limit exploration and insufficient exploitation of the bee colony. Therefore, to balance the exploration and exploitation capabilities of the algorithm, a neighborhood search mechanism with compression factor is proposed by improving search mechanism for the employed bees and onlooker bees. In the new neighborhood search mechanism, the employed bee focuses on expanding the search range and enhancing the global exploration ability, as expressed in Eq. (8). And the onlooker bee focuses on improving the exploitation ability and doing local exploration to obtain better solutions, as shown in Eq. (9).

8 $$v_{ij} = x_{ij} + \phi_{ij} \left( {x_{ij} - x_{kj} } \right) + \psi_{ij}^{1} (x_{best} - x_{ij} )$$

9 $$v_{ij} = cp \times x_{ij} + C \times \phi_{ij} \left( {x_{ij} - x_{kj} } \right) + \psi_{ij}^{2} (x_{best} - x_{ij} )$$

10 $$cp = \frac{2}{{\left| {2 - (C \times mean(\phi_{i,:} ) + \psi_{ij} ) - \sqrt {(C \times mean(\phi_{i,:} ) + \psi_{ij}^{2} )^{2} - 4(C \times mean(\phi_{i,:} ) + \psi_{ij}^{2} )} } \right|}}$$

11 $$C = \frac{1}{{\pi (1{ + }iter^{2} )}}$$

12 $$\phi_{ij} = - 1 + 2z^{B}$$

13 $$\psi_{ij}^{1} = 1.5z^{B}$$

in which, $cp$ is the adaptive compression factor, it can be calculated by Eq. (10), the theoretical value range is [0, 1]; *C* is the Cauchy distribution, calculated by Eq. (11); $\phi_{ij}$ is a uniform random number with values ranging from [− 1, 1], calculated by Eq. (12); $\psi_{ij}^{1}$ is a uniform random number with values ranging from [0, 1.5], which can be calculated by Eq. (13) ; *iter* is the number of generations of the current iteration.

The weight $\psi_{ij}^{2}$ influences the local exploration of the onlooker bees, and then has an impact on the exploitation capacity of the onlooker bees. To test the effect of the parameter $\max \psi_{ij}^{2}$ on the search ability of the onlooker bees, tests are performed with $\max \psi_{ij}^{2}$ = 1.5, 4, 6 and 8 respectively. The test functions are F1, F6, F15 and F25. The colony size is 20, the number of iterations is 1000. Due to their random peculiarity, intelligent heuristic algorithms may generate better or worse solutions than those they anteriorly produced in exploring new solutions. Thus, it is a good select to compare the results by the statistical approach. Then, when testing each function, all algorithms run independently 20 times, the results are shown in Table 2.

**Table 2:** Test results.

Function	$\max \psi_{ij}^{2}$	Mean	Std	Min	Max
F1	1.5	7.1739E-07	2.4133E-06	3.1744E-11	1.0804E-05
	4	4.1221E-15	9.0268E-15	3.1432E-20	2.9755E-14
	6	**3.1039E-18**	**7.1420E-18**	**2.6768E-20**	**3.2539E-17**
	8	4.3654E-16	8.2232E-16	2.6634E-18	2.7075E-15
F6	1.5	0.0011	0.0038	3.2720E-05	0.0170
	4	4.7409E-23	1.9137E-22	5.6182E-29	8.5868E-22
	6	**1.6414E-105**	**7.3404E-105**	**2.2082E-186**	**3.2827E-104**
	8	1.1235E-74	5.0245E-74	6.7342E-143	2.2470E-73
F15	1.5	0.0266	0.0226	1.5299e-13	0.0888
	4	0.0118	0.0149	**0**	0.0395
	6	**0**	**0**	**0**	**0**
	8	**0**	**0**	**0**	**0**
F25	1.5	20.0000	6.4521E-05	19.9997	20.0000
	4	20.0000	2.1087E-09	20.0000	20.0000
	6	1.4211E-15	1.3015E-15	**8.8818E-16**	4.4409E-15
	8	**8.8818E-16**	**0**	**8.8818E-16**	**8.8818E-16**

Significant values are in bold.

As shown in Table 2, it can be seen that $\max \psi_{ij}^{2}$ = 6 in the search formula of the onlooker bee is the most appropriate. Therefore, $\psi_{ij}^{2}$ is a uniform random number with values ranging from [0, 6], which can be calculated by Eq. (14).

14 $$\psi_{ij}^{2} = 6z^{B}$$

To ensure the randomness of exploration, the generation of $\phi_{ij}$ and $\psi_{ij}^{1}$, $\psi_{ij}^{2}$ all introduce Bernoulli chaos mapping. The introduction of *cp* and *C* will further improve the capability of local search.

#### Sustained bees

In traditional ABC algorithm, the colony has three kinds of bees. Due to the mechanism of scout bees, each bee has an upper limit of exploitation, which will lead to some bees may develop to a more optimal solution, but give up exploitation because the upper limit of exploitation has been reached. Therefore, a new bee species is proposed that will continuously exploit the current optimal honey source without the upper limit of exploitation, called sustained bees. Sustained bees are influenced by the global optimal solution to develop new solutions based on the current optimal solution. The update formula of the sustained bee is shown in Eq. (15)

15 $$v_{ij} = x_{iter} + \frac{2}{1 + iter} \cdot rand(x_{best} - x_{iter} ) + \frac{1}{1 + iter} \cdot rand(x_{ij} - x_{kj} )$$

in which, $x_{iter}$ is the current optimal solution.

### A proposed variant of ABC

**Figure 4:**
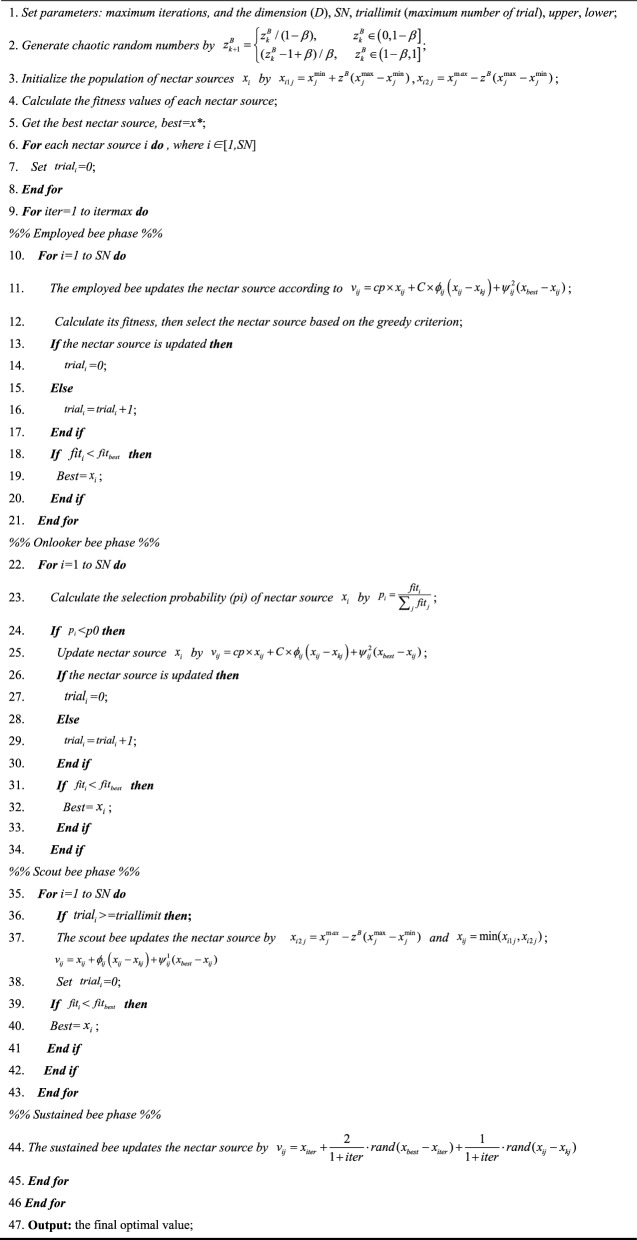
Pseudo-code of CNSABC.

In this study, the traditional ABC algorithm is combined with three improvements, including the Bernoulli chaotic mapping with mutual exclusion mechanism, neighborhood search mechanism with compression factor and sustained bees. Then, a novel chaos and neighborhood search-based ABC algorithm (CNSABC) is formed. Figure 4 shows the pseudo-code of CNSABC.

Time complexity is an important tool to determine the computational complexity of an algorithm. Generally, the time complexity of algorithm is determined by the population size, variable dimensionality and fitness function. In the proposed CNSABC, the population size is *SN* and the dimensionality is *D*. Assuming that the parameter initialization time is *t*_*0*_ and the initialization solution time is *t*_*1*_, this, the time complexity of the initialization phase is shown in Eq. (16).

16 $$T_{1} { = }O\left( {t_{0} + SN \cdot D \cdot t_{1} } \right)$$

In the iterative process, the number of iterations is *K*, and *f(D)* is the time to calculate the fitness value of the optimal individual. The time to select the better individual in the hire in colony is *t*_*2*_, the time to replace the last iteration individual in the employed bee phase is *t*_*3*_, the time to replace the last iteration individual in the onlooker bee phase is *t*_*4*_, the time to replace the last iteration individual in the scout bee phase is* t*_*5*_, the time to replace the last iteration individual in the sustained bee phase is *t*_*6*_, and the time to calculate the weight is *t*_*7*_, so the time complexity of the employed bee phase is Eq. (17 ) is shown.

17 $$T_{2} = O\left( {t_{4} + SN \cdot D \cdot (t_{3} + f(D))} \right)$$

18 $$T_{3} = O\left( {t_{2} + t_{4} + SN \cdot D \cdot (t_{4} + t_{5} + f(D))} \right)$$

19 $$T_{4} = O\left( {D \cdot t_{6} } \right)$$

In conclusion, the time complexity of the CNSABC can be calculated by Eq. (20)

20 $$T = T_{1} + K \cdot (T_{2} + T_{3} + T_{4} )$$

## Experimental results and analyses

In this part, to verify the performance of the presented CNSABC, comprehensive experiments are conducted and analyzed based on the experimental results obtained from 26 benchmark test functions. Firstly, the 26 benchmark test functions are presented and the strategies in Sect. "Improved ABC algorithm" are tested individually to confirm the effectiveness of their improvements. Then CNSABC is compared with five standard algorithms (ABC, PSO, GWO, WOA, and BOA) and finally with five advanced improved ABC (qABC, SBABC, MPGABC, GABC, and NGABC). In addition, all algorithms are coded by Matlab 2020a and run on Intel ® Core i5-2400, CPU@3.0 GHz, 2 GB RAM and Windows 10 computer.

### Benchmark functions

In this experiment, the 26 test functions proposed by Zhong^45^, Luo^46^, Gao^47^, Zhu and Kwong^29^, Karaboga and Akay^48^ are used to test the effectiveness of the presented CNSABC. These test functions include unimodal separable (US) functions (F1, F2, and F3), unimodal non-separable (UN) functions (F4, F5, F6, F7, F8, F9, F10, F11, F12 and F26), multimodal separable (MS) functions (F13, F14, F15, F16, and F17), and multimodal non-separable (MN) functions (F18, F19, F20, F21, F22, F23, F24, and F25). The test functions are shown in Table 3, respectively. Specifically, the unimodal functions can be applied to test the exploitation capability of the algorithm and the multimodal functions can be applied to test the exploration capability.

**Table 3:** Benchmark functions.

Number	Function	Formulation	Minimum	Range	Dimension
F1	Step	$f = \sum\limits_{i = 1}^{n} {\left( {|x_{i} + \frac{1}{2}|} \right)^{2} }$	0	[− 100, 100]	30
F2	Sphere	$f = \sum\limits_{i = 1}^{n} {x_{i}^{2} }$	0	[− 100,100]	50
F3	Sumsquares	$f = \sum\limits_{i = 1}^{n} {ix_{i}^{2} }$	0	[− 100, 100]	100
F4	Beale	$$\begin{aligned} f = & (1.5 - x_{1} + x_{1} x_{2} )^{2} + (2.25 - x_{1} + x_{1} x_{2}^{2} )^{2} \\ & + (2.625 - x_{1} + x_{1} x_{2}^{3} )^{2} \\ \end{aligned}$$	0	[− 4.5, 4.5]	2
F5	Easom	$f = - \cos (x_{1} )\cos (x_{2} )\exp ( - (x_{1} - \pi )^{2} - (x_{2} - \pi )^{2} )$	− 1	[− 100, 100]	2
F6	Matyas	$f = 0.26(x_{1}^{2} + x_{2}^{2} ) - 0.48x_{1} x_{2}$	0	[− 10, 10]	2
F7	Colville	$$\begin{aligned} f = & 100(x_{1}^{2} - x_{2} )^{2} + (x_{1} - 1)^{2} + (x_{3} - 1)^{2} + 90(x_{3}^{2} - x_{4} )^{2} \\ & + 10.1((x_{2} - 1)^{2} + (x_{4} - 1)^{2} ) + 19.8(x_{2} - 1)(x_{4} - 1) \\ \end{aligned}$$	0	[− 10, 10]	4
F8	Trid10	$f = \sum\limits_{i = 1}^{n} {(x_{i} - 1)^{2} - \sum\limits_{i = 2}^{n} {x_{i} x_{i - 1} } }$	− 210	[− 100, 100]	10
F9	Zakharov	$f = \sum\limits_{i = 1}^{n} {x_{i}^{2} + (\sum\limits_{i = 1}^{n} {0.5ix_{i} } )^{2} + } (\sum\limits_{i = 1}^{n} {0.5ix_{i} } )^{4}$	0	[− 5, 10]	10
F10	Powell	$$\begin{aligned} f = & \sum\limits_{i = 1}^{n/4} {((x_{4i - 3} + 10x_{4i - 2} )^{2} + 5(x_{4i - 1} + 10x_{4i} )^{2} } \\ & + (x_{4i - 2} - x_{4i - 1} )^{4} + 10(x_{4i - 3} - x_{4i} )^{4} ) \\ \end{aligned}$$	0	[− 4, 5]	24
F11	Schwefel2.22	$f = \sum\limits_{i = 1}^{n} {|x_{i} |} + \prod\limits_{i = 1}^{n} {|x_{i} |}$	0	[− 10, 10]	30
F12	Schwefel1.2	$f = \sum\limits_{i = 1}^{n} {(\sum\limits_{j = 1}^{i} {x_{j} } )^{2} }$	0	[− 100, 100]	30
F13	Branin	$f = (x_{2} - \frac{5.1}{{4\pi^{2} }}x_{1}^{2} + \frac{5}{\pi }x_{1} - 6)^{2} + 10(1 - \frac{1}{8\pi })\cos (x_{1} ) + 10$	0.398	[− 5,10] × [0,15]	2
F14	Bohachevsky1	$f = x_{1}^{2} + 2x_{2}^{2} - 0.3\cos (3\pi x_{1} ) - 0.4\cos (4\pi x_{2} ) + 0.7$	0	[− 100, 100]	2
F15	Booth	$f = (x_{1} + 2x_{2} - 7)^{2} + (2x_{2} + x_{2} - 5)^{2}$	0	[− 10, 10]	2
F16	Rastrigin	$f = \sum\limits_{i = 1}^{n} {(x_{i}^{2} - 10\cos (2\pi x_{i} ) + 10)}$	0	[− 5.12, 5.12]	30
F17	Michalewicz10	$f = - \sum\limits_{i = 1}^{n} {\sin (x_{i} )(\sin (ix_{i}^{2} /\pi ))^{2m} } ,m = 10$	− 9.6602	$\left[ {0,\pi } \right]$	10
F18	Schaffer	$f = \frac{1}{2} + \frac{{\sin^{2} (\sqrt {x_{1}^{2} + x_{1}^{2} } ) - \frac{1}{2}}}{{(1 + 0.001(x_{1}^{2} + x_{2}^{2} ))^{2} }}$	0	[− 100, 100]	2
F19	Six-hump camel back	$f = 4x_{1}^{2} - 2.1x_{1}^{4} + \frac{1}{3}x_{1}^{6} + x_{1} x_{2} - 4x_{2}^{2} + 4x_{2}^{4}$	− 1.03163	[− 5, 5]	2
F20	Bohachevsky2	$f = x_{1}^{2} + 2x_{2}^{2} - 0.3\cos ((3\pi x_{1} )(4\pi x_{2} )) + 0.3$	0	[− 100, 100]	2
F21	Bohachevsky3	$f = x_{1}^{2} + 2x_{2}^{2} - 0.3\cos (3\pi x_{1} + 4\pi x_{2} ) + 0.3$	0	[− 100, 100]	2
F22	Shubert	$f = \left( {\sum\limits_{i = 1}^{5} {i\cos ((i + 1)x_{1} + i)} } \right) \times \sum\limits_{i = 1}^{5} {i\cos ((i + 1)x_{2} + i)}$	− 186.73	[− 10, 10]	2
F23	Goldstein-price	$$\begin{aligned} f = & (1 + (x_{1} + x_{2} + 1)^{2} \times (19 - 14x_{1} + 3x_{1}^{2} - 14x_{2} + 6x_{1} x_{2} + 3x_{2}^{2} )) \\ & \times (30 + (2x_{1} - 3x_{2} )^{2} \times (18 - 32x_{1} + 12x_{1}^{2} + 48x_{2} \\ & - 36x_{1} x_{2} + 27x_{2}^{2} )) \\ \end{aligned}$$	3	[− 2, 2]	2
F24	Griewank	$f = \frac{1}{4000}\sum\limits_{i = 1}^{n} {x_{i}^{2} - \prod\limits_{i = 1}^{n} {\cos (\frac{{x_{i} }}{\sqrt i })}+1 }$	0	[− 100, 100]	30
F25	Ackely	$f = - 20\exp \left( { - 0.2\sqrt {\frac{1}{n}\sum\limits_{i = 1}^{n} {x_{i}^{2} } } } \right) - \exp \left( {\frac{1}{n}\sum\limits_{i = 1}^{n} {\cos (2\pi x_{i} )} } \right) + 20 + e$	0	[− 100, 100]	30
F26	Rosenbrock	$f = \sum\limits_{i = 1}^{n - 1} {[100(x_{i + 1} - x_{i}^{2} )^{2} + (x_{i} - 1)^{2} ]}$	0	[− 30 to 30]	30

### Influence of improvement points

To determine the effectiveness of the three improvement strategies, each improvement strategy is combined with the ABC algorithm separately to form three variants of ABC. In Table 4, "√" indicates that the improvement strategy is used in combination with ABC, and "●" indicates that the variant of ABC does not use the improvement strategy. In fairness, the same initial parameters are used for all algorithms throughout the testing process, which is a population size of 40, maximum iterations *K* = 1000, and each benchmark function tested 40 times. The experimental results containing the mean, standard deviation, maximum and minimum values are recorded in Table 5. The last row of Table 5 indicates the advantages and disadvantages of this algorithm compared with the ABC, and the symbols " + ", "−" and " = " indicate algorithms that are better than, worse than and equal to other comparisons, respectively.

**Table 4:** ABC with three improvement strategies.

Algorithm	Bernoulli chaotic mapping with mutual exclusion mechanism	neighborhood search mechanism with compression factor	Sustained bees
ABC	●	●	●
CABC	√	●	●
NABC	●	√	●
SABC	●	●	√
CNSABC	√	√	√

**Table 5:** Experimental results of different algorithms.

Function	Method	ABC	CABC	NABC	SABC	CNSABC
F1	Mean	6.3033E−06	2.4282E−06	6.2691E−06	7.0727E−07	**3.1039E−18**
	Std	2.0130E−07	8.6678E−06	2.4807E−07	9.2171E−07	**7.1420E−18**
	Min	5.8446E−06	**5.2019E−22**	5.5784E−06	4.2095E−10	2.6788E−20
	Max	6.6195E−06	3.8698E−05	6.7018E−06	3.1792E−06	**3.2539E−17**
F2	Mean	6.2847E−06	2.8427E−07	6.2504E−06	3.6329E−06	**2.8134E−106**
	Std	1.6352E−07	1.0184E−06	2.2090E−07	1.2866E−06	**1.2582E−105**
	Min	5.9150E−06	4.9370E−16	5.6894E−06	3.4509E−06	**2.0522E−183**
	Max	6.5047E−06	4.5657E−06	6.5750E−06	3.9697E−06	**5.6268E−105**
F3	Mean	3.8452E−07	9.0372E−07	3.9301E−07	1.7948E−07	**3.5800E−120**
	Std	1.2760E−08	1.9426E−06	1.1408E−08	5.2294E−09	**1.6010E−119**
	Min	3.6005E−07	2.4119E−20	3.5951E−07	1.7174E−07	**1.1430E−184**
	Max	4.1262E−07	6.9222E−06	4.0789E−07	1.8984E−07	**7.1598E−119**
F4	Mean	6.1324E−06	2.6211E−10	6.1705E−06	1.1225E−16	**0**
	Std	2.4487E−07	1.0600E−09	2.9880E−07	6.5506E−18	**0**
	Min	5.5697E−06	3.7583E−23	5.7279E−06	1.0225E−16	**0**
	Max	6.4864E−06	4.7611E−09	6.6895E−06	1.2185E−16	**0**
F5	Mean	− **1**	− **1**	− **1**	− **1**	− **1**
	Std	**0**	**0**	**0**	**0**	**0**
	Min	− **1**	− **1**	− **1**	− **1**	− **1**
	Max	− **1**	− **1**	− **1**	− **1**	− **1**
F6	Mean	8.7270E−07	2.6850E−13	**0**	1.0455E−07	1.0201E−185
	Std	3.8989E−06	5.8987E−13	**0**	2.7715E−09	**0**
	Min	1.4819E−19	1.3942E−22	**0**	1.0115E−04	**0**
	Max	1.7437E−05	2.3341E−12	**0**	1.1115E−04	2.0401E−184
F7	Mean	5.2501E−07	1.3380	**5.2499E−07**	1.0493E−07	1.5580E−06
	Std	3.0373E−08	1.6399	**2.4749E−08**	2.6622E−09	5.2187E−06
	Min	4.7029E−07	0.0149	4.8010E−07	1.0021E−07	**4.4525E−19**
	Max	5.9022E−07	5.6029	**5.6770E−07**	1.1001E−04	2.2125E−05
F8	Mean	1.0396E−05	− 207.8724	1.0465E−05	1.0493E−05	− **210**
	Std	7.3214E−07	3.6546	7.9215E−07	2.6622E−07	**1.9702E−07**
	Min	8.9681E−05	− 209.9708	9.0461E−06	1.0021E−05	− **210**
	Max	1.2311E−05	− 197.5812	1.1759E−05	1.1001E−05	− **210**
F9	Mean	1.1097E−16	8.5507E−06	1.0945E−16	1.9789e−16	**6.2715E−100**
	Std	4.6669E−17	3.8240E−05	2.4950E−17	2.8963E−19	**2.3746E−99**
	Min	1.7438E−17	4.4038E−38	7.5005E−17	1.9752E−16	**1.7799E−221**
	Max	2.1300E−16	1.7101E−04	1.7141E−16	1.9836E−016	**1.0534E−98**
F10	Mean	5.2868E−06	7.5294E−05	5.2579E−06	1.1426e−06	**1.6414E−105**
	Std	2.8452E−07	1.5559E−04	3.4197E−07	5.6568e−08	**7.3404E−105**
	Min	4.4538E−06	8.5998E−07	4.3805E−06	1.0516e−06	**2.2082E−186**
	Max	5.7277E−06	6.6871E−04	5.7764E−06	1.2878e−06	**3.2827E−104**
F11	Mean	4.3656E−24	0.0846	3.3958E−24	1.1427E−24	**1.3537E−69**
	Std	2.7450E−24	0.1390	2.9587E−24	6.2568E−26	**5.7278E−69**
	Min	6.9525E−25	2.7524E−04	1.0237E−25	1.0427E−24	**3.0989E−109**
	Max	1.0785E−23	0.4288	1.1501E−23	1.3483E−024	**2.5651E−68**
F12	Mean	6.3405E−08	5.8752E−06	6.1543E−08	2.4233e−08	**7.8687E−96**
	Std	9.5820E−09	1.8559E−05	8.7716E−09	1.2772e−09	**3.5135E−95**
	Min	4.3485E−08	8.2522E−20	4.5237E−08	2.2418e−08	**4.7330E−168**
	Max	8.3883E−08	7.8695E−05	8.2976E−08	2.7763e−08	**1.5714E−94**
F13	Mean	0.4414	0.3979	0.4026	0.3999	**0.3979**
	Std	0.1945	7.7581E−12	0.0213	0.0016	**0**
	Min	0.3979	0.3979	0.3979	0.3979	**0.3979**
	Max	1.2678	0.3979	0.4931	0.4020	**0.3979**
F14	Mean	3.4222E−05	**0**	3.4178E−05	3.4134e−05	**0**
	Std	5.2493E−07	**0**	3.9572E−07	4.5806e−07	**0**
	Min	3.3466E−05	**0**	3.3521E−05	3.3517e−05	**0**
	Max	3.5216E−05	**0**	3.5194E−05	3.5337e−05	**0**
F15	Mean	4.0092E−04	1.7244E−18	4.0260E−04	5.5868E−06	**0**
	Std	6.1776E−06	6.6668E−18	8.7173E−06	1.1948E−07	**0**
	Min	3.8881E−04	**0**	3.8854E−05	5.4143E−06	**0**
	Max	4.1116E−04	2.9805E−17	4.1873E−04	5.8971E−06	**0**
F16	Mean	0.0013	108.1361	0.0013	**3.6110E−05**	35.0225
	Std	2.7345E−05	32.1619	3.4933E−05	**9.8445E−07**	34.0596
	Min	0.0013	58.1761	0.0013	3.4373E−05	**0**
	Max	0.0014	191.2156	0.0014	**3.8131E−05**	113.4248
F17	Mean	− 8.6629	− **8.7732**	− 6.2679	− 8.2530	− 8.6220
	Std	0.4138	0.5350	0.7127	**0.2102**	0.5782
	Min	− 9.2661	− **9.5363**	− 8.1738	− 8.8003	− 9.4654
	Max	− 7.7728	− 7.5645	− 5.0401	− **7.9181**	− 7.4623
F18	Mean	0.0074	0.0085	0.0044	0.0081	**0.0029**
	Std	0.0032	0.0022	0.0050	**0.0018**	0.0046
	Min	1.2892E−04	0.0040	**0**	0.0025	**0**
	Max	**0.0097**	**0.0097**	**0.0097**	0.0098	**0.0097**
F19	Mean	− **1.0316**	− **1.0316**	− **1.0316**	− **1.0316**	− **1.0316**
	Std	1.9729E−16	2.2781E−16	**1.5282E−16**	1.5571E−16	1.9729E−16
	Min	− **1.0316**	− **1.0316**	− **1.0316**	− **1.0316**	− **1.0316**
	Max	− **1.0316**	− **1.0316**	− **1.0316**	− **1.0316**	− **1.0316**
F20	Mean	3.4469E−05	**0**	3.4433E−05	3.4236E−05	**0**
	Std	5.4295E−07	**0**	4.9410E−07	5.4052E−07	**0**
	Min	3.3548E−05	**0**	3.3876E−05	3.3456E−05	**0**
	Max	3.5764E−05	**0**	3.5459E−05	3.5210E−05	**0**
F21	Mean	3.4264E−05	2.2430E−08	3.4143E−05	3.4185E−06	**0**
	Std	4.6562E−07	9.5144E−08	5.5772E−07	5.7542E−08	**0**
	Min	3.3440E−05	**0**	3.3348E−05	3.3422E−06	**0**
	Max	3.5247E−05	4.2609E−07	3.5811E−05	3.5498E−06	**0**
F22	Mean	− **186.7309**	− **186.7309**	− **186.7309**	− **186.7309**	− **186.7309**
	Std	1.7251E−14	3.4503E−14	4.2757E−14	**4.3395E−15**	2.6082E−14
	Min	− **186.7309**	− **186.7309**	− **186.7309**	− **186.7309**	− **186.7309**
	Max	− **186.7309**	− **186.7309**	− **186.7309**	− **186.7309**	− **186.7309**
F23	Mean	**3**	**3**	**3**	**3**	**3**
	Std	1.7109E−15	1.6205E−15	1.7967E−15	**2.8054E−21**	1.0830E−15
	Min	**3**	**3**	**3**	**3**	**3**
	Max	**3**	**3**	**3**	**3**	**3**
F24	Mean	0.0238	0.0536	0.0239	0.0179	**0**
	Std	8.6652E−04	0.1067	7.9754E−04	0.0097	**0**
	Min	0.0220	3.8566E−05	0.0227	0.0057	**0**
	Max	0.0254	0.4665	0.0251	0.0453	**0**
F25	Mean	0.0433	20.4410	0.0433	0.0428	**1.4211E−15**
	Std	4.4771E−05	0.1549	4.3436E−05	7.2692E−06	**1.3015E−15**
	Min	0.0432	20.0200	0.0432	0.0428	**8.8818E−16**
	Max	0.0434	20.6306	0.0436	0.0429	**4.4409E−15**
F26	Mean	1.0886E−09	1.0826E−09	1.070E−09	1.0816E−09	**1.0628E−09**
	Std	6.6009E−11	**5.2912E−11**	6.1933E−11	8.0853E−11	5.8918E−11
	Min	9.3998E−10	9.9561E−10	9.6418E−10	**9.3590E−11**	9.6195E−10
	Max	1.1698E−09	1.1841E−09	1.1454E−09	1.2034E−09	**1.1382E−09**
	+ / = /−		13/1/12	15/1/10	20/1/4	22/1/3

Significant values are in bold.

By comparing the mean and standard deviation in Table 5, it can be analyzed that sustained bees and Bernoulli chaotic mapping with mutual exclusion mechanism have limited improvement on ABC and performs poorly in the tests of the 4 benchmark functions F7, F11, F16 and F25; the neighborhood search mechanism with compression factor has stronger improvement on ABC; and the CNSABC shows stronger exploration and exploitation when the three improved strategies work simultaneously. In the benchmark functions F4, F5, F13, F14, F15, F20, F21 and F24 are extremely close to the theoretical optimal values, due to the fact that the values exceed the display digits of Matlab, thus, the decimal digits are not displayed. The performance of each algorithm in the tests of benchmark functions F5, F19 and F23 is not extremely different. In addition, each comparison algorithm is compared with the CNSABC for 5% nonparametric statistics Wilcoxon test and Friedman test, the *p*-values of Wilcoxon test for ABC, CABC, NABC and SABC are 1.5856E−07, 8.3922E−09, 1.3414E−10 and 1.6690E−08 respectively, which are less than 5%, indicating that there are remarkable distinctions between algorithms. Friedman test is shown in Table 6, Mean-rank represents the average ranking of each algorithm, and smaller values represent better algorithm performance. In conclusion, the three proposed mechanisms, including the Bernoulli chaotic mapping with mutual exclusion mechanism, a neighborhood search mechanism with compression factor and sustained bees, have superiority to improve the performance of ACO.

**Table 6:** Mean rank of Friedman test between CNSABC and comparison algorithm.

Algorithm	ABC	CABC	NABC	SABC	CNSABC
Mean-rank	3.6106	3.3317	3.4663	2.8365	1.7548

### Comparison with other advanced original algorithms

To verify the advantage of the CNSABC, five commonly used metaheuristic optimization algorithms including PSO, ABC, GWO, WOA and BOA are used for comparison. The parameters of different algorithms are set to be same: population size *N* = 20, maximum iterations *K* = 1000, and each benchmark function tested 40 times. The other parameters are set based on the recommended values in the original manuscript of the literature, and the mean, standard deviation, minimum and maximum values of the results of the 40 experiments are counted in Table 7. In Table 7, " + ", "−" and " = " indicate the number of benchmark functions in which the CNSABC is better, worse and equal to other original algorithms or the number of benchmark functions in which other original algorithms are better, worse and equal to the CNSABC among the 26 benchmark functions, respectively.

**Table 7:** Comparison results of the CNSABC and other algorithms.

Function	Method	PSO	GWO	WOA	BOA	ABC	CNSABC
F1	Mean	9.9983E−04	0.3769	1.0014	6.1614	6.3033E−06	**3.1039E−18**
	Std	0.0010	0.2935	0.4301	0.5447	2.0130E−07	**7.1420E−18**
	Min	3.2640E−05	5.6646E−06	0.1215	5.2255	5.8446E−06	**2.6788E−20**
	Max	0.0044	0.9795	1.7686	6.9756	6.6195E−06	**3.2539E−17**
F2	Mean	9.8093E−04	1.3071E−49	1.6781E−64	1.8775E−14	6.2847E−06	**2.8134E−106**
	Std	0.0011	3.0155E−49	7.5047E−64	9.4745E−16	1.6352E−07	**1.2582E−105**
	Min	7.6959E−05	2.3794E−52	3.7087E−89	1.7181E−14	5.9150E−06	**2.0522E−183**
	Max	0.0041	1.3420E−48	3.3562E−63	2.0525E−14	6.5047E−06	**5.6268E−105**
F3	Mean	0.0105	7.0063E−49	3.6869E−68	1.9082E−14	3.8452E−07	**3.5800E−120**
	Std	0.0151	9.5299E−49	1.0679E−67	8.2889E−16	1.2760E−08	**1.6010E−119**
	Min	9.4001E−04	7.4803E−51	5.8433E−89	1.7631E−14	3.6005E−07	**1.1430E−184**
	Max	0.0701	3.9225E−48	4.4130E−67	2.0350E−14	4.1262E−07	**7.1598E−119**
F4	Mean	**0**	0.1524	0.1030	0.2452	6.1324E−06	**0**
	Std	**0**	0.3127	0.2117	0.2672	2.4487E−07	**0**
	Min	**0**	1.7376E−10	3.2949E−14	5.4218E−05	5.5697E−06	**0**
	Max	**0**	0.7621	0.5462	0.7995	6.4864E−06	**0**
F5	Mean	**− 1**	**− **1.0000	**− **0.9999	**− **1.0000	**− 1**	**− 1**
	Std	**0**	1.2955E−07	5.0303E−04	1.3492E−07	**0**	**0**
	Min	**− 1**	**− **1.0000	**− **1.0000	**− **1.0000	**− 1**	**− 1**
	Max	**− 1**	**− **1.0000	**− **0.9977	**− **1.0000	**− 1**	**− 1**
F6	Mean	8.2400E−50	2.1966E−155	**2.9546E−269**	1.6753E−15	8.7270E−07	1.0201E−185
	Std	3.0783E−49	9.8237E−155	**0**	5.4816E−16	3.8989E−06	0
	Min	8.8250E−54	8.3014E−179	**1.6593E−308**	9.2805E−16	1.4819E−19	0
	Max	1.3873E−48	4.3933E−154	**5.9092E−268**	2.7288E−15	1.7437E−05	2.0401E−184
F7	Mean	**0**	1.1141	1.9480	2.6390	5.2501E−07	1.5580E−06
	Std	**0**	1.0348	2.4746	3.7073	3.0373E−08	5.2187E−06
	Min	**0**	5.9414E−04	6.6754E−05	0.0537	4.7029E−07	4.4525E−19
	Max	**0**	3.1913	8.8697	15.4071	5.9022E−07	2.2125E−05
F8	Mean	**− 210**	**− **150.7897	**− **159.0448	**− **157.7900	1.0396E−05	**− 210**
	Std	**6.8573E−13**	65.9035	57.9565	61.4902	7.3214E−07	1.9702E−07
	Min	**− 210**	**− **209.9954	**− **209.6166	**− **209.9668	8.9681E−05	**− 210**
	Max	**− 210**	**− **29.8692	**− **44.8485	**− **35.7384	1.2311E−05	**−210**
F9	Mean	2.4717E−29	4.0931E−56	3.4033	1.6830E−14	1.1097E−16	**6.2715E−100**
	Std	4.0564E−29	1.4443E−55	8.2487	1.3291E−15	4.6669E−17	**2.3746E−99**
	Min	4.7573E−31	8.2220E−61	3.1438E−10	1.5052E−14	1.7438E−17	**1.7799E−221**
	Max	1.3828E−28	6.5005E−55	37.5228	1.9781E−14	2.1300E−16	**1.0534E−98**
F10	Mean	0.6772	1.8821E−37	5.0775E−30	1.8012E−14	5.2868E−06	**1.6414E−105**
	Std	0.6083	7.9132E−37	2.1359E−29	1.0547E−15	2.8452E−07	**7.3404E−105**
	Min	0.0307	2.2312E−43	1.7346E−123	1.5293E−14	4.4538E−06	**2.2082E−186**
	Max	2.1964	3.5477E−36	9.54E647E−29	2.0615E−14	5.7277E−06	**3.2827E−104**
F11	Mean	1.3650	1.1620E−29	**4.3341E−95**	1.0385E−11	4.3656E−24	1.3537E−69
	Std	0.7271	7.2151E−30	**1.9208E−94**	3.2983E−12	2.7450E−24	5.7278E−69
	Min	0.3853	1.8066E−30	5.6007E−106	6.1034E−13	6.9525E−25	**3.0989E−109**
	Max	3.2849	2.4250E−29	**8.5940E−94**	1.2420E−11	1.0785E−23	2.5651E−68
F12	Mean	1.4708	3.4813E−11	3.0270E+04	1.8846E−14	6.3405E−08	**7.8687E−96**
	Std	1.2305	1.2836E−10	1.2502E+04	1.3422E−15	9.5820E−09	**3.5135E−95**
	Min	0.1846	2.7505E−16	8.0609E+03	1.6636E−14	4.3485E−08	**4.7330E−168**
	Max	4.8010	5.7304E−10	5.1101E+04	2.1998E−14	8.3883E−08	**1.5714E−94**
F13	Mean	**0.3979**	0.3982	0.3995	0.4011	0.4414	**0.3979**
	Std	**0**	0.0015	0.0056	0.0035	0.1945	**0**
	Min	**0.3979**	0.3979	0.3979	0.3980	0.3979	**0.3979**
	Max	**0.3979**	0.4046	0.4223	0.4094	1.2678	**0.3979**
F14	Mean	**0**	**0**	**0**	0.1245	3.4222E−05	**0**
	Std	**0**	**0**	**0**	0.1952	5.2493E−07	**0**
	Min	**0**	**0**	**0**	0	3.3466E−05	**0**
	Max	**0**	**0**	**0**	0.4208	3.5216E−05	**0**
F15	Mean	**0**	7.2520E−08	4.2942E−04	0.1176	4.0092E−04	**0**
	Std	**0**	6.1077E−08	3.6752E−04	0.2171	6.1776E−06	**0**
	Min	**0**	1.4146E−09	9.4707E−06	1.6045E−04	3.8881E−04	**0**
	Max	**0**	2.1881E−07	0.0011	0.7806	4.1116E−04	**0**
F16	Mean	33.9462	1.2985	**0**	26.8966	0.0013	35.0225
	Std	6.0792	3.6228	**0**	66.6190	2.7345E−05	34.0596
	Min	22.2727	**0**	**0**	**0**	0.0013	**0**
	Max	44.8553	15.3599	**0**	218.7261	0.0014	113.4248
F17	Mean	**− 9.5382**	**− **7.5901	**− **6.4129	**− **7.1242	**− **8.6629	**− **8.6220
	Std	**0.1001**	0.9132	1.1306	1.2718	0.4138	0.5782
	Min	**− 9.6552**	**− **9.3386	**− **8.5491	**− **8.8675	**− **9.2661	**− **9.4654
	Max	**− 9.2601**	**− **5.5938	**− **4.7952	**− **4.7608	**− **7.7728	**− **7.4623
F18	Mean	**0**	0.0039	0.0044	0.0109	0.0074	0.0029
	Std	**0**	0.0049	0.0050	0.0066	0.0032	0.0046
	Min	**0**	**0**	**0**	4.4761E−07	1.2892E−04	**0**
	Max	**0**	0.0097	0.0096	0.0372	0.0097	0.0097
F19	Mean	**− 1.0316**	**− 1.0316**	**− 1.0316**	**− 1.0316**	**− 1.0316**	**− 1.0316**
	Std	2.2781E−16	2.5408E−09	3.9702E−12	3.9759E−11	1.9789E−16	**1.9729E−16**
	Min	**− 1.0316**	**− 1.0316**	**− 1.0316**	**− 1.0316**	**− 1.0316**	**− 1.0316**
	Max	**− 1.0316**	**− 1.0316**	**− 1.0316**	**− 1.0316**	**− 1.0316**	**− 1.0316**
F20	Mean	**0**	**0**	**0**	5.9971E−14	3.4469E−05	**0**
	Std	**0**	**0**	**0**	2.6719E−13	5.4295E−07	**0**
	Min	**0**	**0**	**0**	**0**	3.3548E−05	**0**
	Max	**0**	**0**	**0**	1.1952E−12	3.5764E−05	**0**
F21	Mean	**0**	**0**	8.9345E−15	0.0113	3.4264E−05	**0**
	Std	**0**	**0**	1.9096E−14	0.0506	4.6562E−07	**0**
	Min	**0**	**0**	5.5511E−16	**0**	3.3440E−05	**0**
	Max	**0**	**0**	8.3267E−14	0.2264	3.5247E−05	**0**
F22	Mean	**− 186.7309**	**− **186.7095	**− **186.7246	**− **186.7279	**− 186.7309**	**− 186.7309**
	Std	2.0619E−14	0.0955	0.0196	0.0131	**1.7251E−14**	2.6082E−14
	Min	**− 186.7309**	**− 186.7309**	**− 186.7309**	**− 186.7309**	**− 186.7309**	**− 186.7309**
	Max	**− 186.7309**	**− **186.3036	**− **186.6449	**− **186.6722	**− 186.7309**	**− 186.7309**
F23	Mean	**3.0000**	**3.0000**	3.0003	3.0625	**3.0000**	**3.0000**
	Std	**3.8120E−16**	3.2807E−05	6.1539E−04	0.1134	1.7109E−15	1.0830E−15
	Min	**3.0000**	**3.0000**	**3.0000**	3.0018	**3.0000**	**3.0000**
	Max	**3.0000**	**3.0000**	3.0026	3.4922	**3.0000**	**3.0000**
F24	Mean	0.0030	1.2212E−06	5.5511E−18	**0**	0.0238	**0**
	Std	0.0046	3.4172E−17	2.4825E−14	**0**	8.6652E−04	**0**
	Min	1.8222E−07	1.1102E−16	**0**	**0**	0.0220	**0**
	Max	0.0099	2.2204E−16	1.1102E−16	**0**	0.0254	**0**
F25	Mean	19.3147	20.8670	12.4117	1.4193E−13	0.0433	**1.4211E−15**
	Std	3.7745	0.1209	10.3982	4.9531E−13	4.4771E−05	**1.3015E−15**
	Min	3.2850	20.4993	**8.8818E−16**	**8.8818E−16**	0.0432	**8.8818E−16**
	Max	20.4522	21.0077	20.9472	2.2320E−12	0.0434	**4.4409E−15**
F26	Mean	98.3532	26.6086	27.6165	28.9464	1.0886E−09	**1.0628E−09**
	Std	122.4866	0.5504	0.6782	0.0315	6.6009E−11	**5.8918E−11**
	Min	27.0493	25.8600	26.5105	28.8615	**9.3998E−10**	9.6195E−10
	Max	414.7268	27.1976	28.7339	28.9837	1.1698E−09	**1.1382E−09**
	+ / = /−	5/7/14	0/3/23	3/2/21	0/1/25	1/1/24	10/7/9

Significant values are in bold.

Specifically, for the results of the mean, CNSABC gets the best results in 20 out of 26 benchmark functions (F1, F2, F3, F4, F5, F8, F9, F10, F12, F13, F14, F15, F19, F20, F21, F22, F23, F24, F25 and F26); For the standard deviation, CNSABC obtains the best results in 18 functions (F1, F2, F3, F4, F5, F9, F10, F12, F13, F14, F15, F19, F20, F21, F22, F24, F25 and F26). This can indicate that the CNSABC has a strong exploitation capability and stability. Meanwhile, CNSABC obtains the optimal result for the minimum value among 22 functions (F1, F2, F3, F4, F5, F8, F9, F10, F11, F12, F13, F14, F15, F16, F18, F19, F20, F21, F22, F23, F24 and F25). The result indicates that the CNSABC is the best performance among the compared algorithms.

Figure 6 shows representative convergence curves for only a subset of the 26 benchmark functions (F1, F3, F6, F10, F13, F15, F19, and F25)^49^. To allow for a more intuitive comparison, the* y*-axis of the convergence curves is the logarithm of the fitness, besides F19 function.

From the results in Table 7, it can be seen that the CNSABC demonstrates excellent performance of other functions in the tests of F2, F3, F4, F9, F12, F19, F25 and F26, however, the worst performance in the tests of F16 and F17.

**Figure 5:**
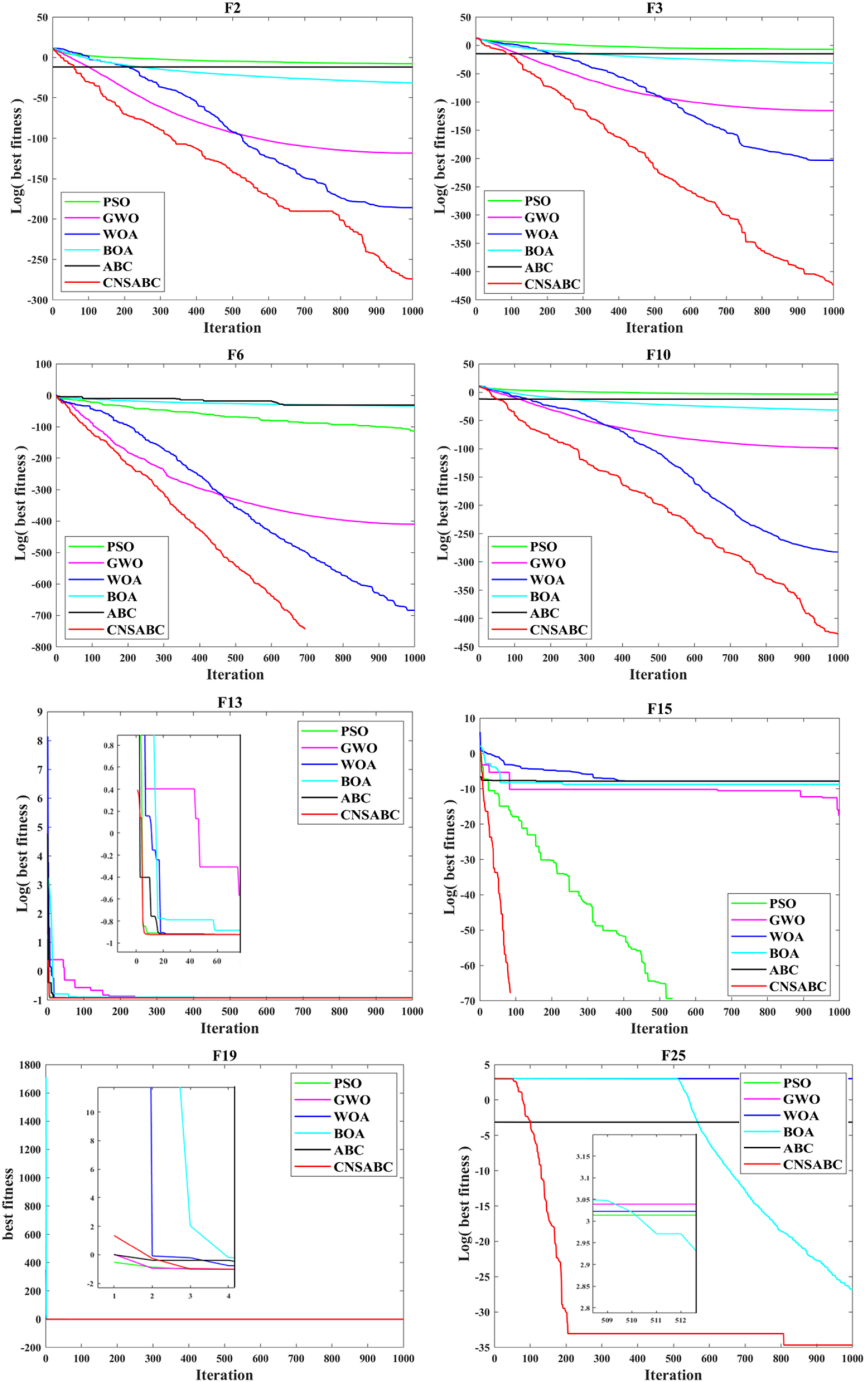
A subset of the 26 benchmark functions.

In Fig. 5, the convergence curves show different characteristics for different search strategies, one is a slow convergence from the beginning of the iteration, and the other is a cliff-like decline in the convergence process. The first kind of curve mainly reflected in the single-peaked benchmark functions, which are F1–F14. And the second kind of curve mainly reflected in the multimodal benchmark functions, which are F15–F25. By analyzing the different types of benchmark functions, the CNSABC has excellent performance in both convergence accuracy and convergence process. Most convergence curves are the first kind of convergence curves, reflecting that the CNSABC achieves a well balance between exploitation capability and exploration capability. Each comparison algorithm is compared with CNSABC for 5% nonparametric statistics Wilcoxon test and Friedman test. *p*-values of Wilcoxon test for PSO, GWO, WOA BOA and ABC are 7.2866E−04, 5.4838E−11, 3.4412E−11, 5.3564E−15, 2 and 1.2364E−07, respectively, which are less than 5%, indicating that there are remarkable distinctions between algorithms. Friedman test can be seen in Table 8, Mean-rank represents the average rank of each algorithm, the smaller the value is, the wonderful performance of the algorithm is.

**Table 8:** Mean rank of Friedman test between the CNSABC and comparison algorithm.

Algorithm	PSO	GWO	WOA	BOA	ABC	CNSABC
Mean-rank	3.3702	3.3942	3.7260	4.5577	3.9760	1.9760

### Comparison with other improved ABC algorithms

In this part, the performance of the CNSABC is compared with other improved ABC algorithms in 26 benchmark function tests, including qABC, SBABC, MPGABC, GABC and NGABC. To be fair, the different algorithms all contain parameters with the same settings: population size *N* = 20, maximum iterations *K* = 1000, and 40 tests for each benchmark function. The experimental results are recorded in Table 9, containing the mean, standard deviation, minimum and maximum values of the results obtained from the 40 sets of experiments. in the last row of Table 9, " + ", "−" and " = " indicate the amount of benchmark functions in which the CNSABC is better, worse and equal to other algorithms or the number of benchmark functions in which other algorithms are better. In Table 9, in terms of the mean, the CNSABC obtains the best results for 21 of the 26 benchmark functions (F2, F3, F4, F5, F6, F8, F9, F10, F11, F12, F13, F14, F15, F18, F19, F20, F21, F22, F23, F24 and F25). In terms of the minimum value, the CNSABC obtains the best results for 22 functions (F2, F3, F4, F5, F6, F8, F9, F10, F11, F12, F13, F14, F15, F16, F18, F19, F20, F21, F22, F23, F24 and F25). The results indicate that CNSABC has wonderful performance in terms of the convergence accuracy. Figure 7 shows representative convergence curves for only a subset of the 26 benchmark functions (F2, F3, F6, F10, F13, F15, F19, and F25).

**Table 9:** Comparison results of CNSABC with other improved ABC algorithms.

Function	Method	qABC	SBABC	MPGABC	GABC	NGABC	CNSABC
F1	Mean	3.7701E−09	6.6365E−07	3.1193E−15	**6.6354E−23**	3.4543E−015	3.1039E−18
	Std	5.2168E−09	1.3684E−06	1.1804E−14	**2.9370E−22**	1.9876E−15	7.1420E−18
	Min	3.3988E−12	1.2303E−10	5.3618E−31	**0**	1.7383E−15	2.6788E−20
	Max	1.6819E−08	5.6812E−06	5.2493E−14	**1.3141E−21**	8.1986E−14	3.2539E−17
F2	Mean	4.3092E−09	8.7041E−07	8.6589E−14	1.6537E−30	1.78628E−56	**2.8134E−106**
	Std	9.0077E−09	2.1330E−06	3.8239E−13	7.1395E−30	1.8961E−55	**1.2582E−105**
	Min	2.6257E−11	8.4017E−12	3.9298E−31	1.9237E−62	4.4695E−58	**2.0522E−183**
	Max	4.0153E−08	9.2649E−06	1.7111E−12	3.1977E−29	7.7772E−55	**5.6268E−105**
F3	Mean	5.5987E−08	4.2390E−07	2.3551E−07	1.5862E−22	4.7565E−65	**3.5800E−120**
	Std	2.2723E−07	8.8494E−07	1.0532E−06	7.0932E−22	4.8456E−65	**1.6010E−119**
	Min	2.3464E−12	4.4758E−12	1.5453E−25	8.2603E−65	5.7945E−66	**1.1430E−184**
	Max	1.0207E−06	3.7020E−06	4.7101E−06	3.1722E−21	1.7956E−64	**7.1598E−119**
F4	Mean	4.0948E−12	2.1071E−17	5.3203E−22	**0**	**0**	**0**
	Std	1.2932E−11	5.7959E−17	2.2122E−21	**0**	**0**	**0**
	Min	1.3588E−19	6.0222E−27	**0**	**0**	**0**	**0**
	Max	5.6142E−11	2.0481E−16	9.9048E−21	**0**	**0**	**0**
F5	Mean	− 1.0000	− **1**	− **1**	− **1**	− **1**	− **1**
	Std	7.4623E−12	**0**	**0**	**0**	**0**	**0**
	Min	− **1**	− **1**	− **1**	− **1**	− **1**	− **1**
	Max	− 1.0000	− **1**	− **1**	− **1**	− **1**	− **1**
F6	Mean	2.7511E−15	2.8202E−13	1.9093E−30	2.5724E−79	6.7954E−85	**1.0201E−185**
	Std	8.5456E−15	1.0041E−12	7.2614E−30	1.1504E−78	1.9465E−84	**0**
	Min	6.1589E−26	2.7173E−25	1.8368E−80	1.1016E−281	1.8156E−81	**0**
	Max	3.5771E−14	4.5042E−12	3.2240E−29	5.1447E−78	4.6411E−84	**2.0401E−184**
F7	Mean	0.5746	0.0630	2.0793	2.1884E−13	**1.4949E−20**	1.5580E−06
	Std	1.0984	0.0641	2.3790	9.3941E−13	**6.4512E−20**	5.2187E−06
	Min	3.8387E−05	6.3952E−04	0.0067	**1.2664E−28**	5.6133E−25	4.4525E−19
	Max	3.7575	0.2401	6.2430	4.2068E−12	4.4612E−19	2.2125E−05
F8	Mean	− 196.6313	− 202.1253	− 125.8798	− 209.9177	− 209.9891	− **210**
	Std	20.6028	4.2484	91.1910	0.3681	0.6356	**1.9702E−07**
	Min	− 209.9413	− 209.5711	− 209.8860	− 210.0000	− 210.0000	− **210**
	Max	− 125.6782	− 195.0126	104.6546	− 208.3540	− 208.2669	− **210**
F9	Mean	4.6618E−07	1.3870E−22	3.1163E−06	3.6932E−13	5.9362E−08	**6.2715E−100**
	Std	6.0021E−07	6.1964E−22	1.0901E−05	1.0685E−12	7.4937E−08	**2.3746E−99**
	Min	2.8179E−09	2.6939E−42	2.3260E−15	1.4392E−29	6.7929E−10	**1.7799E−221**
	Max	1.7034E−06	2.7712E−21	4.7594E−05	4.6337E−12	2.8698E−07	**1.0534E−98**
F10	Mean	1.3742E−07	4.5084E−09	3.4429E−06	1.3901E−24	1.7952E−29	**1.6414E−105**
	Std	5.2419E−07	1.1359E−08	9.8772E−06	5.8775E−24	3.6545E−29	**7.3404E−105**
	Min	2.6016E−11	1.0221E−12	5.8097E−16	7.9166E−55	2.3456E−33	**2.2082E−186**
	Max	2.3591E−06	5.5341E−08	3.7053E−05	2.6327E−23	1.4632E−28	**3.2827E−104**
F11	Mean	8.1024E−11	6.6529E−08	2.7650E−06	3.4100E−14	1.4656E−26	**1.3537E−69**
	Std	1.6759E−10	1.3906E−07	1.1449E−05	1.4446E−13	8.4686E−27	**5.7278E−69**
	Min	5.1714E−15	5.7698E−11	2.3264E−19	1.5578E−29	8.8966E−28	**3.0989E−109**
	Max	7.4338E−10	5.8208E−07	5.1277E−05	6.4696E−13	1.4895E−26	**2.5651E−68**
F12	Mean	4.8146E−15	1.3552E−09	9.0755E−05	4.0173E−10	1.3046E−35	**7.8687E−96**
	Std	1.1672E−14	3.0411E−09	2.6462E−04	1.6336E−09	1.4946E−35	**3.5135E−95**
	Min	3.9866E−24	2.2273E−15	6.9077E−17	1.0403E−21	2.7943E−37	**4.7330E−168**
	Max	3.6598E−14	1.0878E−08	0.0011	7.3229E−09	1.6461E−34	**1.5714E−94**
F13	Mean	**0.3979**	**0.3979**	**0.3979**	0.4481	**0.3979**	**0.3979**
	Std	2.2152E−11	2.1305E−12	1.0129E−12	0.2246	5.9273E−11	**0**
	Min	**0.3979**	**0.3979**	**0.3979**	**0.3979**	**0.3979**	**0.3979**
	Max	**0.3979**	**0.3979**	**0.3979**	1.4021	**0.3979**	**0.3979**
F14	Mean	1.9929E−15	1.3323E−16	**0**	**0**	**0**	**0**
	Std	8.4043E−15	4.6353E−16	**0**	**0**	**0**	**0**
	Min	**0**	**0**	**0**	**0**	**0**	**0**
	Max	3.7637E−14	1.9984E−15	**0**	**0**	**0**	**0**
F15	Mean	2.9510E−15	2.5340E−17	1.4199E−30	**0**	**0**	**0**
	Std	8.9795E−15	6.8421E−17	6.3502E−30	**0**	**0**	**0**
	Min	1.0584E−25	1.5419E−24	**0**	**0**	**0**	**0**
	Max	3.8145E−14	2.5409E−16	2.8399E−29	**0**	**0**	**0**
F16	Mean	1.0670	1.1672	2.4883	19.8034	**0.1510**	35.0225
	Std	0.9281	1.1977	1.6942	8.6505	**0.3638**	34.0596
	Min	1.8414E−06	0.0176	8.6846E−12	3.9798	1.7844E−08	**0**
	Max	2.8782	3.8803	5.9697	36.8844	**0.9950**	113.4248
F17	Mean	− 8.1298	− 7.0662	− 7.8566	− 7.6571	− **8.7885**	− 8.6220
	Std	**0.5463**	0.5550	0.8761	0.8766	0.5851	0.5782
	Min	− 9.3489	− 8.2570	− 9.3191	− 9.0374	− **9.5109**	− 9.4654
	Max	− 7.3769	− 6.1315	− 6.2440	− 5.9947	− **7.6272**	− 7.4623
F18	Mean	0.0034	0.0058	0.0058	0.0063	**0.0023**	0.0029
	Std	0.0039	0.0038	0.0049	0.0048	**0.0036**	0.0046
	Min	4.4636E−09	7.4315E−05	**0**	**0**	5.159E−12	**0**
	Max	**0.0097**	**0.0097**	**0.0097**	**0.0097**	**0.0097**	**0.0097**
F19	Mean	− **1.0316**	− **1.0316**	− **1.0316**	− **1.0316**	− **1.0316**	− **1.0316**
	Std	1.1391E−16	1.2478E−16	2.1612E−16	2.0376E−16	**5.0941E−17**	1.9729E−16
	Min	− **1.0316**	− **1.0316**	− **1.0316**	− **1.0316**	− **1.0316**	− **1.0316**
	Max	− **1.0316**	− **1.0316**	− **1.0316**	− **1.0316**	− **1.0316**	− **1.0316**
F20	Mean	**0**	**0**	**0**	**0**	**0**	**0**
	Std	**0**	**0**	**0**	**0**	**0**	**0**
	Min	**0**	**0**	**0**	**0**	**0**	**0**
	Max	**0**	**0**	**0**	**0**	**0**	**0**
F21	Mean	6.0345E−12	9.2288E−11	1.6653E−17	**0**	**0**	**0**
	Std	1.7879E−11	3.9088E−10	7.4476E−17	**0**	**0**	**0**
	Min	**0**	**0**	**0**	**0**	**0**	**0**
	Max	7.7451E−11	1.7519E−09	3.3307E−16	**0**	0.0000	**0**
F22	Mean	− **186.7309**	− **186.7287**	− **186.7309**	− **186.7309**	− **186.7309**	− **186.7309**
	Std	5.2322E−09	0.0076	3.6304E−14	2.9160E−13	1.0003E−13	**2.6082E−14**
	Min	− 186.7309	− 186.7309	− 186.7309	− 186.7309	− 186.7309	− **186.7309**
	Max	− 186.7309	− 186.6970	− 186.7309	− 186.7309	− 186.7309	− **186.7309**
F23	Mean	**3.0000**	**3.0000**	**3.0000**	**3.0000**	**3.0000**	**3.0000**
	Std	5.4561E−15	2.6194E−15	**1.0289E−15**	1.5282E−15	1.4998E−15	1.0830E−15
	Min	**3.0000**	**3.0000**	**3.0000**	**3.0000**	**3.0000**	**3.0000**
	Max	**3.0000**	**3.0000**	**3.0000**	**3.0000**	**3.0000**	**3.0000**
F24	Mean	3.6314E−05	1.8264E−05	4.9323E−04	0.0020	1.4649E−09	**0**
	Std	8.1499E−05	2.5328E−05	0.0022	0.00040	3.6469E−09	**0**
	Min	1.4316E−09	4.1881E−07	**0**	**0**	5.4689E−013	**0**
	Max	3.3578E−04	9.0629E−05	0.0099	0.0099	4.6979E−09	**0**
F25	Mean	8.9528E−09	0.0066	2.0970E−12	2.8422E−15	1.4686E−15	**1.4211E−15**
	Std	2.2524E−08	0.0039	9.3707E−12	1.8134E−15	1.9686E−15	**1.3015E−15**
	Min	4.4409E−15	0.0021	9.0165E−16	8.8916E−16	**7.4964E−17**	8.8818E−16
	Max	8.7009E−08	0.0160	4.1909E−11	5.4309E−15	6.7965E−15	**4.4409E−15**
F26	Mean	1.0568e−05	2.8731	0.2456	1.9846E−04	1.5963	**1.0628E−09**
	Std	1.6598e−05	5.9110	0.2495	1.4562E−04	0.0265	**5.8918E−11**
	Min	8.9564e−06	6.0440E−04	0.0195	9.4698E−05	1.4268	**9.6195E−10**
	Max	2.6546e−05	21.8394	0.8654	3.6597E−04	1.9634	**1.1382E−09**
	+ / = / = −	1/5/20	1/6/19	1/7/18	1/6/19	3/6/17	12/10/4

Significant values are in bold.

In Table 9, the CNSABC is stronger than other improved ABC algorithms, whether it is a single-peak benchmark function or a multimodal benchmark function, which also reflects the exploration and development capability of the CNSABC to achieve a well balance.

**Figure 6:**
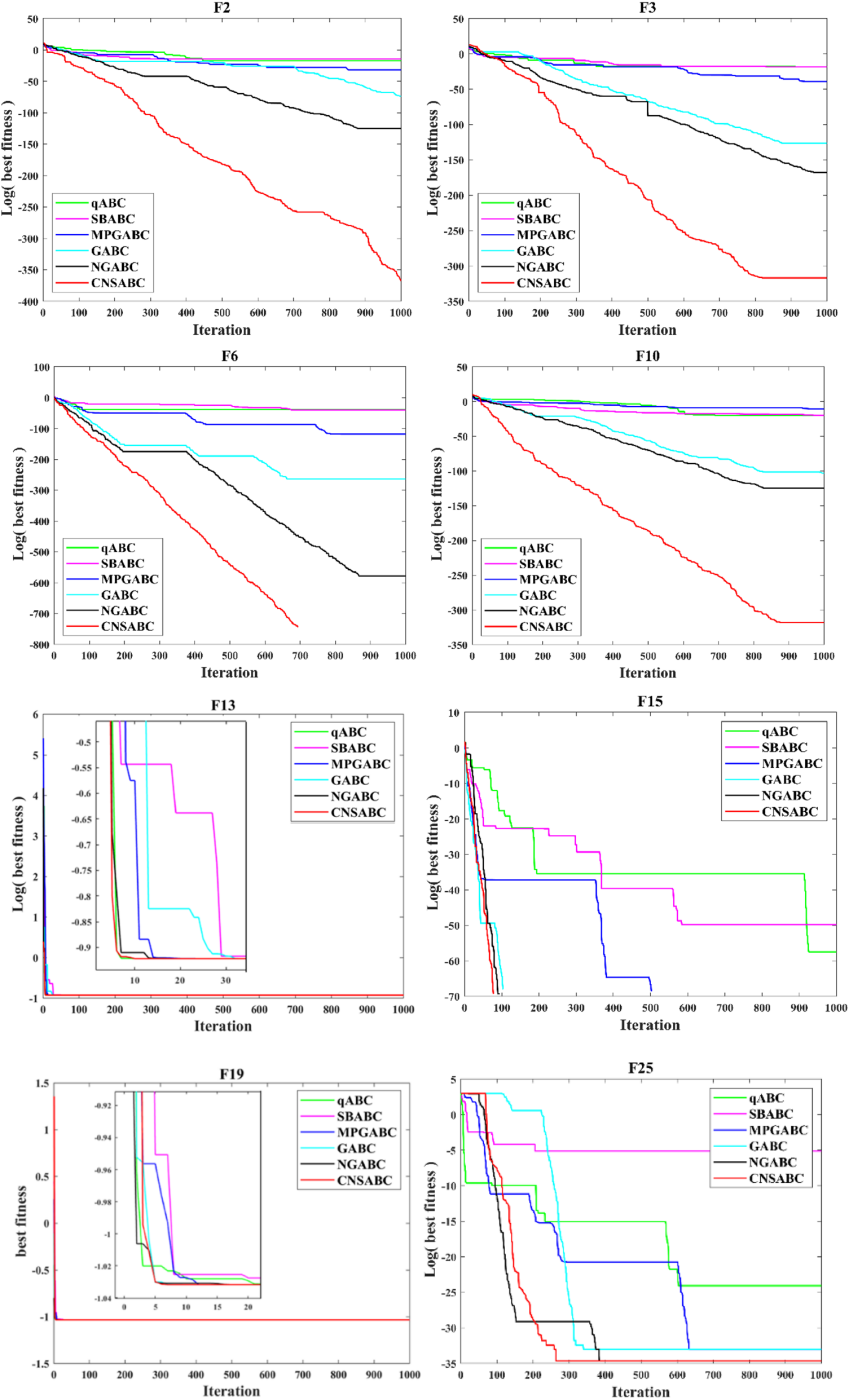
A subset of the 26 benchmark functions.

In Fig. 6, the CNSABC has outstanding superiority compared with other improved ABC algorithms. In most of the convergence curves show a slow decreasing trend from the beginning of the iterations, but have been better in fitness than other algorithms, which indicates that the CNSABC has a strong exploration and exploitation capability. This is attributed to the introduction of three mechanisms. Each comparison algorithm is compared with CNSABC for 5% nonparametric statistics Wilcoxon test and Friedman test. *p*-values of Wilcoxon test for qABC, SBABC, MPGABC GABC and NGABC are 1.0921E−09, 6.2631E−10, 7.0176E−10, 1.8958E−05 and 1.2600E−02 respectively, Moreover, the better results of CNSABC also can be demonstrated by Friedman test in Table 10.

**Table 10:** Mean rank of Friedman test between CNSABC and comparison algorithms.

Algorithm	qABC	SBABC	MPGABC	GABC	NGABC	CNSABC
Mean-rank	4.2404	4.5915	4.2067	3.2404	2.6010	2.1202

## CNSABC for solving engineering optimization problems

### Tension/compression spring design optimization problem

The main objective of this engineering problem is to minimize the mass of the tension/compression spring. The optimization constraints of this problem are described as follows:

1. Shear stress.
2. Surge frequency.
3. Minimum deflection.

**Figure 7:**
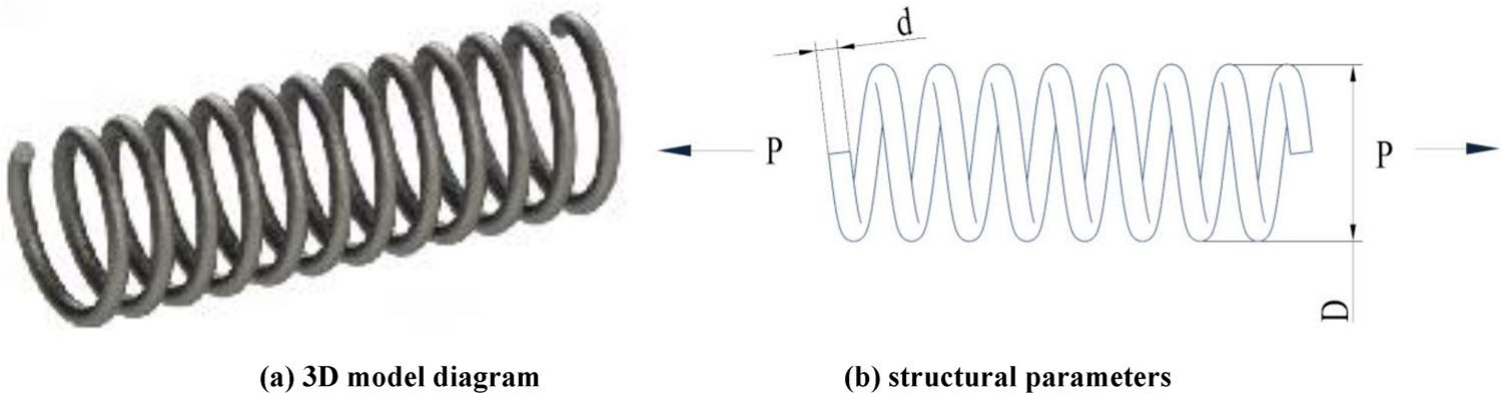
Schematic view of tension–compression spring design.

The schematic diagram of spring is exhibit in Fig. 7

.

This problem has three variables: wire diameter (*d*), mean coil diameter (*D*), and the number of active coils (P). The mathematical model is described as follows:

Consider:

$${\vec{\text{x}}} = [x_{1} \, x_{2} \, x_{3} ] = [d \, D \, P]$$

Minimize:

$$f(\vec{x}) = (x_{3} + 2)x_{2} x_{1}^{2}$$

Subject to:

$$\begin{gathered} g_{1} \left( {\vec{x}} \right) = 1 - \frac{{x_{2}^{3} x_{3} }}{{71785x_{1}^{4} }} \le 0 \hfill \\ g_{2} \left( {\vec{x}} \right) = \frac{{4x_{2}^{2} - x_{1} x_{2} }}{{12566\left( {x_{2} x_{1}^{3} - x_{1}^{4} } \right)}} + \frac{1}{{5108x_{1}^{2} }} \le 0 \hfill \\ g_{3} \left( {\vec{x}} \right) = 1 - \frac{{140.45x_{1} }}{{x_{2}^{2} x_{3} }} \le 0 \hfill \\ g_{4} \left( {\vec{x}} \right) = \frac{{x_{1} + x_{2} }}{1.5} - 1 \le 0 \hfill \\ \end{gathered}$$

$$0.05 \le x_{1} \le 2.0, \, 0.25 \le x_{1} \le 1.3, \, 2.0 \le x_{1} \le 15.0$$

**Figure 8:**
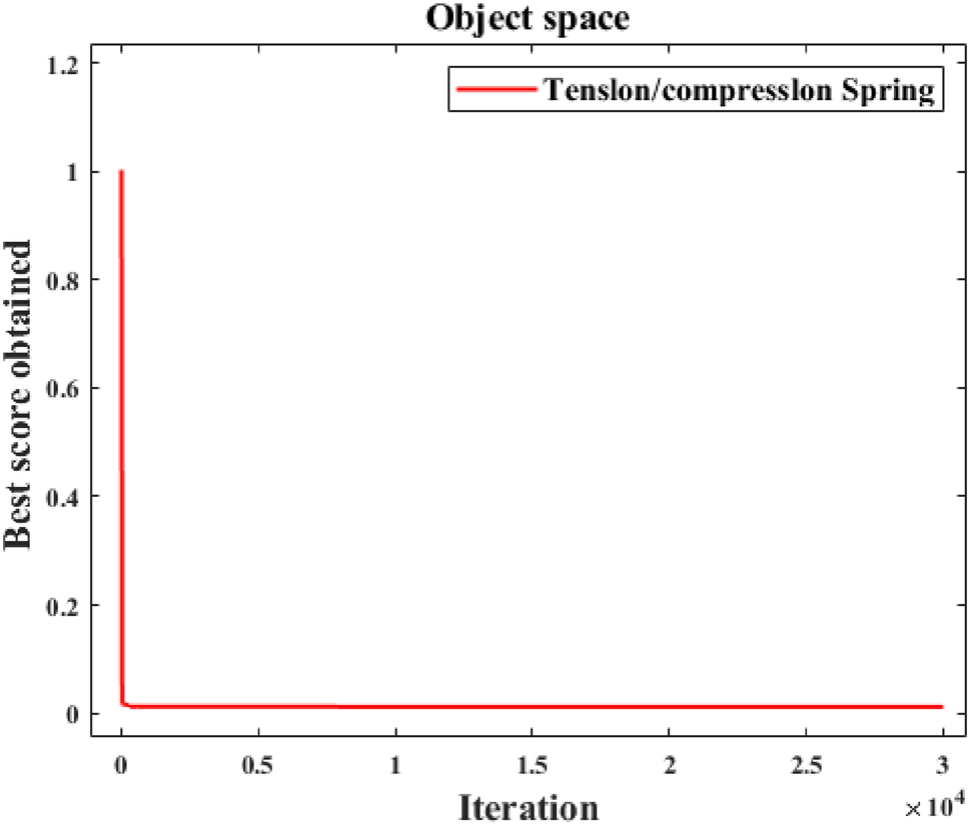
Convergence analysis of CNSABC for tension/compression spring design.

In fairness, CNSABC uses the same penalty function as the other algorithms, the results are shown in Table 11. Table 12 shows the mean, standard deviation, minimum and maximum values of the 10 experiments of the CNSABC. Figure 8 shows the adaptation convergence curve of CNSABC computing the tension/compression spring design. The best solution is obtained by CNSABC at design variables $\vec{x} = [x_{1} \, x_{2} \, x_{3} ]$ with $f(\vec{x}) = {0}{\text{.012192037027776}}$. In solving tension/compression spring design problem, results show that the optimal weights compared with EPO, SHO, GWO, MVO, SCA, EPO, DE, ES, GA, RO, improved HS, HSCA, CB-ABC and I-ABC greedy, CNSABC increased by 3.6735%, 3.8028%, 3.8356%, 4.87755%, 4.0727%, 3.6728%, 3.7739%, 3.8559%, 4.0630%, 3.8392%, 3.7770%, 3.3734%, 3.3734%, and 3.3734%, respectively. CNSABC has superiority performance than the other algorithms.

**Table 11:** Comparison results for tension/compression spring design.

Algorithms	Optimal values for variables			Optimum weight
	*d*	*D*	*P*	
CNSABC	0.051097	0.348205	11.4769346	**0.012192037**
EPO^52^	0.051087	0.342908	12.0898	0.012656987
SHO^53^	0.051144	0.343751	12.0955	0.012674
GWO	0.050178	0.341541	12.07349	0.012678321
MVO^54^	0.05	0.315956	14.22623	0.01281693
SCA^55^	0.05078	0.334779	12.72269	0.012709667
EPO^56^	0.051087	0.342908	12.0898	0.0126569
DE^57^	0.051609	0.354714	11.410831	0.0126702
ES^58^	0.051989	0.363965	10.890522	0.012681
GA	0.05148	0.351661	11.632201	0.0127048
RO^59^	0.05137	0.349096	11.76279	0.0126788
Improved HS^60^	0.051154	0.349871	12.076432	0.0126706
HSCA^61^	–	–	–	0.12665
CB-ABC	–	–	–	0.12665
I-ABC greedy	–	–	–	0.12665

Significant values are in bold.

**Table 12:** Statistical results obtained from CNSABC for tension/compression spring design.

Algorithm	Mean	Std	Min	Max
CNSABC	0.012210562	1.46875E−05	0.012192037	0.012245703

### Speed reducer design optimization problem

**Figure 9:**
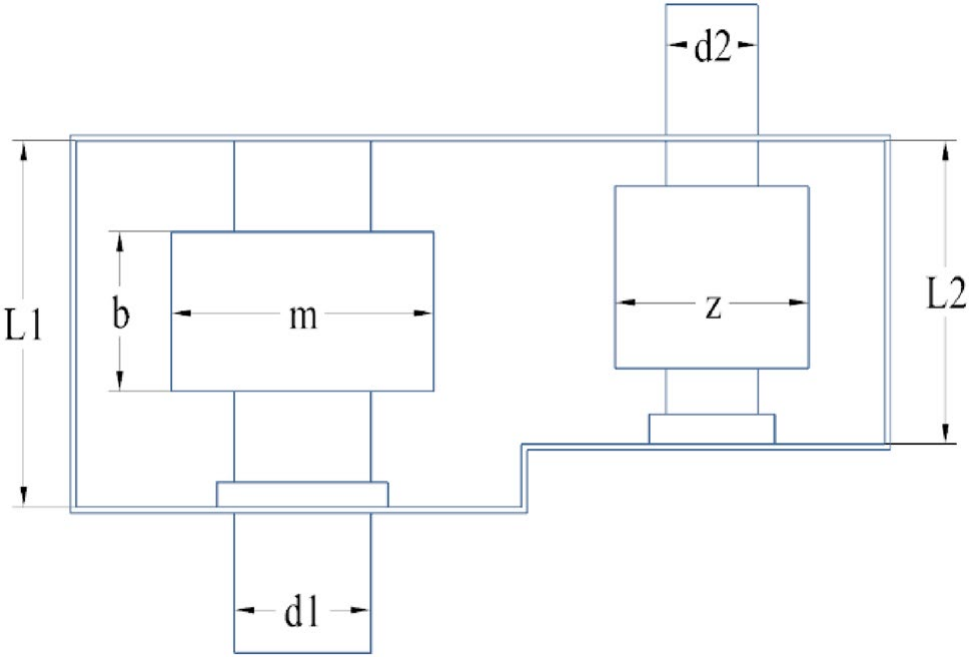
structural parameters.

The main objective of this engineering problem is to minimize the mass of the reducer as much as possible. There are 7 design variables in this model, which are the face width (*b)*, the tooth die (*m*), the number of pinion teeth (*p*), the length of the first and second shaft between the bearings (*l*_*1*_), (*l*_*2*_), the diameter of the first shaft (*d*_*1*_) and the diameter of the second shaft (*d*_*2*_). The design variables of the reducer are reflected in Fig. 9. The mathematical model can be described as:

Consider:

$$\vec{x} = [x_{1} \, x_{2} \, x_{3} \, x_{4} \, x_{5} \, x_{6} \, x_{7} \left] = \right[b \, m \, p \, l_{1} \, l_{2} \, d_{1} \, d_{2} ]$$

Minimize:

$$\begin{aligned} f\left( {\vec{x}} \right) = & 0.7854x_{1} x_{2}^{2} \left( {3.3333x_{3}^{2} + 14.9334x_{3} - 43.0934} \right) - 1.508x_{1} \left( {x_{6}^{2} + x_{7}^{2} } \right) + 7.4777\left( {x_{6}^{3} + x_{7}^{3} } \right) \\ & + 0.7854\left( {x_{4} x_{6}^{2} + x_{5} x_{7}^{2} } \right) \\ \end{aligned}$$

Subject to:

$$\begin{gathered} g_{1} \left( {\vec{x}} \right) = \frac{27}{{x_{1} x_{2}^{2} x_{3} }} - 1 \le 0 \hfill \\ g_{2} \left( {\vec{x}} \right) = \frac{397.5}{{x_{1} x_{2}^{2} x_{3}^{2} }} - 1 \le 0 \hfill \\ g_{3} \left( {\vec{x}} \right) = \frac{{1.93x_{4}^{3} }}{{x_{2} x_{6}^{4} x_{3} }} - 1 \le 0 \hfill \\ g_{4} \left( {\vec{x}} \right) = \frac{{1.93x_{5}^{3} }}{{x_{2} x_{7}^{4} x_{3} }} - 1 \le 0 \hfill \\ g_{5} \left( {\vec{x}} \right) = \frac{{\left[ {\left( {745\left( {x_{4} /x_{2} x_{3} } \right)} \right)^{2} + 16.9 \times 10^{6} } \right]^{1/2} }}{{110x_{6}^{3} }} - 1 \le 0 \hfill \\ \end{gathered}$$

$$\begin{gathered} g_{6} \left( {\vec{x}} \right) = \frac{{\left[ {\left( {745\left( {x_{5} /x_{2} x_{3} } \right)} \right)^{2} + 157.5 \times 10^{6} } \right]^{1/2} }}{{85x_{7}^{3} }} - 1 \le 0 \hfill \\ g_{7} \left( {\vec{x}} \right) = \frac{{x_{2} x_{3} }}{40} - 1 \le 0 \hfill \\ g_{8} \left( {\vec{x}} \right) = \frac{{5x_{2} }}{{x_{1} }} - 1 \le 0 \hfill \\ g_{9} \left( {\vec{x}} \right) = \frac{{x_{1} }}{{12x_{2} }} - 1 \le 0 \hfill \\ g_{10} \left( {\vec{x}} \right) = \frac{{1.5x_{6} + 1.9}}{{x_{4} }} - 1 \le 0 \hfill \\ g_{11} \left( {\vec{x}} \right) = \frac{{1.1x_{7} + 1.9}}{{x_{5} }} - 1 \le 0 \hfill \\ \end{gathered}$$

in which:

$$\begin{gathered} 2.6 \le x_{1} \le 3.6,0.7 \le x_{2} \le 0.8,17 \le x_{3} \le 28,7.3 \le x_{4} \le 8.3 \\ 7.3 \le x_{5} \le 8.3,2.9 \le x_{6} \le 3.9,5.0 \le x_{7} \le 5.5 \\ \end{gathered}$$

**Figure 10:**
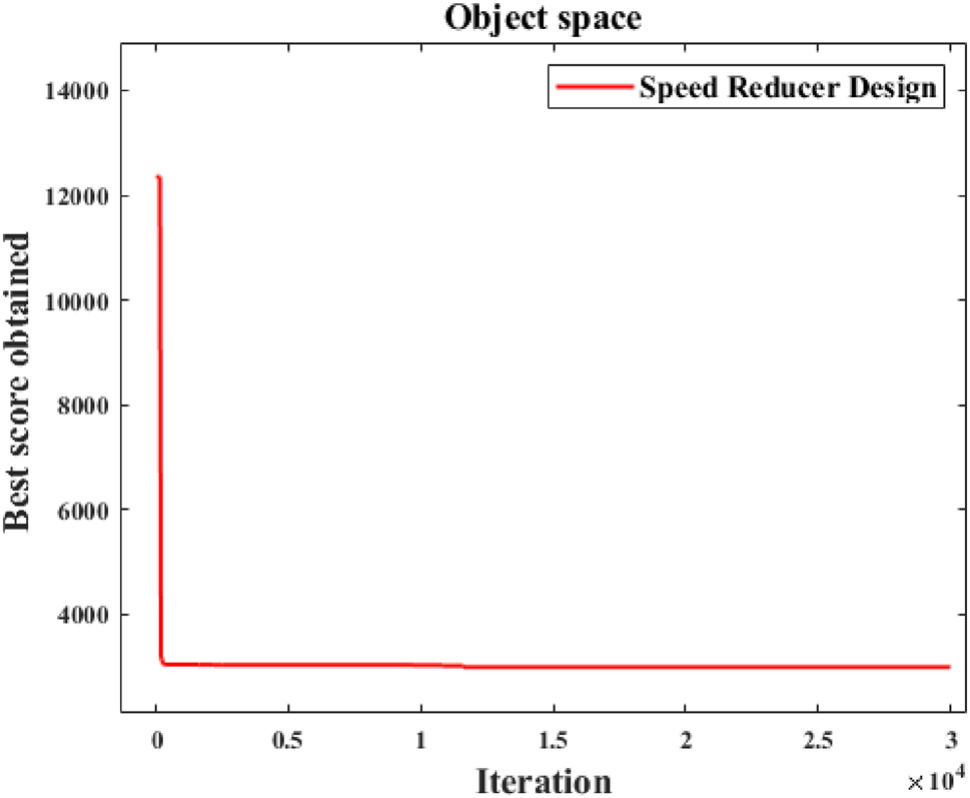
Convergence analysis of CNSABC for speed reducer design.

In fairness, CNSABC uses the same penalty function as the other algorithms, the results of CNSABC and the other algorithms are displayed in Table 13^50^^,^^51^. Table 14 shows the mean, standard deviation, minimum and maximum values of the 10 experiments of the CNSABC. Figure 10 shows the adaptation convergence curve of CNSABC computing the tension/compression spring design problem. The best solution is obtained by CNSABC at design variables $\vec{x} = [x_{1} \, x_{2} \, x_{3} \, x_{4} \, x_{5} \, x_{6} \, x_{7} ]$ with objective function $f(\vec{x}) = 2994.534574$. The optimal value obtained in speed reducer design problem, CNSABC improved over SHO, GWO, POS, MVO, SCA, GSA, GA, AO, AOA, HS FA, HSCA, CB-ABC and I-ABC greedy by 0.13605%, 0.2271%, 0.3757%, 0.2816%, 1.1909%, 1.8566%, 2.3828%, 0.4409%, 0.1149%, 1.1400%, 0.5205%, 1.3358e-09, 1.3358e-09 and 2.2041e-08. CNSABC has wonderful performance than the other algorithms.

**Table 13:** Comparison results for speed reducer design.

Algorithms	Optimal values for variables							Optimum weight
	*b*	*m*	*p*	$l_{1}$	$l_{2}$	$d_{1}$	$d_{2}$	
CNSABC	3.50000	0.7	17	7.30000007	7.7153376	3.350349	5.286599	**2994.471066**
SHO	3.50159	0.7	17	7.3	7.8	3.35127	5.28874	2998.5507
GWO	3.50669	0.7	17	7.380933	7.815726	3.357847	5.286768	3001.288
PSO	3.500019	0.7	17	8.3	7.8	3.352412	5.286715	3005.763
MVO	3.508502	0.7	17	7.392843	7.816034	3.358073	5.286777	3002.928
SCA	3.508755	0.7	17	7.3	7.8	3.46102	5.289213	3030.563
GSA	3.6	0.7	17	8.3	7.8	3.369658	5.289224	3051.12
GA	3.510253	0.7	17	8.35	7.8	3.362201	5.287723	3067.561
AO^50^	3.5021	0.7	17	7.3099	7.7476	3.3641	5.2994	3007.7328
AOA	3.50384	0.7	17	7.3	7.72933	3.35649	5.2867	2997.9157
HS	3.52012	0.7	17	8.37	7.8	3.36697	5.28871	3029.002
FA^51^	3.50749	0.7001	17	7.71967	8.08085	3.35151	5.28705	3010.13749
HSCA	–	–	–	–	–	–	–	2994.47107
CB-ABC	–	–	–	–	–	–	–	2994.47107
I-ABC greedy	–	–	–	–	–	–	–	2994.4710

Significant values are in bold.

**Table 14:** Statistical results obtained from CNSABC for speed reducer design.

Algorithm	Mean	Std	Min	Max
CNSABC	2995.362975	0.986396417	2994.471066	2997.28675

## Conclusions

In this study, a chaotic and neighborhood search-based ABC algorithm (CNSABC) is presented to solve the shortcomings of traditional ABC for solving optimization problems. Firstly, Bernoulli chaos mapping with mutual exclusion mechanism is proposed to increase the diversity of populations and strengthen the global exploration capability. Secondly, neighborhood search mechanism with compression factor and sustained bees are presented to improve the local exploration and exploitation capability, and further to avoid the appearance of premature maturity. Subsequently, three groups of simulation experiments based on 26 benchmark functions are conducted to compare the CNSABC with eight existing variants of ABC and five commonly used metaheuristic optimization algorithms. The experimental results composed of “Mean”, “Std.”, “Max”, “Min” of the 26 benchmark functions verify the dominant of CNSABC in the optimal solution search ability. In detail, the overall performance of CNSABC is generally superior to PSO, ABC, GWO, WOA and BOA on 21, 26, 23 and 26 out of 26 functions. For the five variants of ABC, the CNSABC outperforms qABC, SBABC, MPGABC, GABC and NGABC with 26 benchmark functions of 25, 25, 25, 22 and 24, respectively. Finally, the CNSABC is applied to two engineering examples, the experimental results show that CNSABC can effectively solve practical application problems.

Although the proposed CNSABC achieves excellent results in terms of exploitation capability and local exploration capability, the research of the CNSABC is still in the initial stage, and many problems need further study, such as a deficiency of low computational efficiency. Future work will focus on further enhancing the algorithm efficiency and exploring more improvement directions. For example, search strategies can be borrowed and combined with other algorithms. The application is extended to more practical applications, such as PID parameter optimization, and improved parameter search combined with neural network.

## Data Availability

The datasets used during the current study available from the corresponding author on reasonable request.
